# *Antennaria dioica* (L.) Gaertn. (Asteraceae): From Metabolite Profiling to Comprehensive Evaluation of In Vitro and In Silico Enzyme Inhibitory Activity

**DOI:** 10.1002/fsn3.71854

**Published:** 2026-05-13

**Authors:** Dimitrina Zheleva‐Dimitrova, Gokhan Zengin, Iglika Lessigiarska, Ivanka Tsakovska, Radostina Nikolova‐Kejova, Ivayla Zheleva‐Kyuchukova, Georgi Momekov, Reneta Gevrenova

**Affiliations:** ^1^ Department of Pharmacognosy, Faculty of Pharmacy Medical University Sofia Bulgaria; ^2^ Physiology and Biochemistry Research Laboratory, Department of Biology, Science Faculty Selcuk University Konya Turkey; ^3^ Institute of Biophysics and Biomedical Engineering Bulgarian Academy of Sciences Sofia Bulgaria; ^4^ Acibadem City Clinic UMBAL Tokuda Sofia Bulgaria; ^5^ Department of Pharmacology, Pharmacotherapy and Toxicology, Faculty of Pharmacy Medical University Sofia Bulgaria

**Keywords:** *ADME/Tox*, *Antennaria dioica (L.) Gaertn*, *docking*, *leontopodic acids*, *UHPLC‐HRMS/MS*

## Abstract

*Antennaria dioica* (L.) Gaertn., (tribe Gnaphalieae of Asteraceae family) is known in traditional medicine for treatment of biliary and respiratory ailments, and for its astringent and hemostatic properties. This work aimed at in‐depth phytochemical profiling and in vitro antioxidant and enzyme inhibition assessment of methanol‐aqueous extract from the *A. dioica* aerial parts integrated with molecular docking study. An ultra‐high‐performance liquid chromatography coupled to high resolution tandem mass spectrometry (UHPLC‐HRMS/MS) analysis revealed a total of 130 secondary metabolites including 39 acylquinic acids, 26 caffeoylhexaric acids, 34 carboxylic and phenolic acids and coumarins, as well as 31 flavonoids. For the first time, 68 core structures alongside caffeoylglucaric derivatives leontopodic acid A and B are reported in the species. The *A. dioica* extract inhibited *α*‐amylase and *α*‐glucosidase (0.45 mmol and 0.11 mmol ACAE/g, respectively), and acetylcholinesterase (AChE) and butyrylcholinesterase (BChE) (1.93 and 1.14 mmol Galantamine equivalent per gram extract (GALE/g), respectively). The extract exhibited antioxidant activity in different in vitro assays. In search for bioactive compounds, the extract's identified molecules were docked within four target enzymes: AChE (PDB code 4EY7), BChE (PDB code 7AMZ), *α*‐amylase (PDB code 1Z32), and *α*‐glucosidase (PDB code 5NN6). Twenty three core structures showed favorable docking scores for *α*‐amylase and *α*‐glucosidase, while only 4 of them were relevant for AChE and BChE. Methylbutyryl‐ and *p*‐hydroxybenzoyl‐tricaffeoylglucaric acid alongside apigenin 7‐*O*‐caffeoylglucoside were highlighted to contain potential ligands of *α*‐amylase and *α*‐glucosidase. The Absorption, Distribution, Metabolism, Excretion, and Toxicity (ADME/Tox) properties of the selected compounds were evaluated in silico. Apigenin 7‐*O*‐caffeoyl‐*β*‐glucoside was outlined as having good potential for interaction with *α*‐amylase and *α*‐glucosidase, and favorable ADME/Tox properties.

## Graphical abstract

*Antennaria dioica* (L.) Gaertn. is a perennial herb from the tribe Gnaphalieae of the Asteraceae family. In‐depth phytochemical profiling and in vitro antioxidant and enzyme inhibition assessment of methanol‐aqueous extract from the *A. dioica* aerial parts were integrated with molecular docking study. An ultra‐high‐performance liquid chromatography coupled to high resolution tandem mass spectrometry (UHPLC‐HRMS/MS) analysis revealed a total of 130 secondary metabolites. The compounds identified in the extract were investigated with *in silico* approaches for their potential to bind AChE and BChE, as well as *α*‐amylase and *α*‐glucosidase. Their ADME/Tox properties were also evaluated. The extract was found to contain more potential ligands of *α*‐amylase and *α*‐glucosidase than of AChE and BChE in accordance with the use of the plant in traditional medicine. Among the compounds, apigenin 7‐*O*‐caffeoyl‐*β*‐glucoside was outlined as having good potential for interaction with *α*‐amylase and *α*‐glucosidase, and favorable ADME/Tox properties.

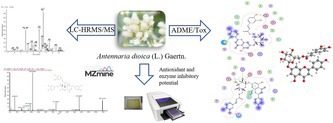

## 1 Introduction

*Antennaria dioica* (L.) Gaertn. is a perennial herb from the tribe Gnaphalieae of the Asteraceae family, distributed across Europe and Asia in dry grasslands, meadows, and rocky terrains (Babotă et al. 2018; Basaraba et al. 2022). *A. dioica* is commonly referred to as mountain everlasting or cat's foot. In Europe, the flowering heads have been used in an ethnopharmacological approach as an anti‐inflammatory, choleretic, diuretic, and antioxidant agent, which aligns with its reputation as a source of phenolic constituents (Babotă et al. 2018; Basaraba et al. 2022).

As far as we know, there is not a thorough assessment of the secondary metabolites in the plant, and fewer studies have been reported for the active compounds of the species with Romanian and Ukrainian provenance. Thus, the total phenolic and flavonoid contents reached 36.27 mg gallic acid equivalent per gram dry weight (GAE/g dw) and 21.88 mg Quercetin equivalent per gram dry weight (QE/g dw), respectively, in the ethanol‐aqueous extracts of flowering heads (Babotă et al. 2018). The authors determined by HPLC–MS up to 502.70 ± 25.11 mg/100 g dw chlorogenic acid and 448.48 ± 22.22 mg/100 g dw quercitrin in the methanolic and hydroethanolic extracts, respectively, together with kaempferol, apigenin, hispidulin and *β*‐sitosterol. Interestingly, the aerial parts of *A. dioica* from Ukrainian origin reveled 164.5 ± 0.17 μm/g dw plant material isoquercitrin (not found in the aforementioned study) and 944.48 ± 0.22 μm/g dw rosmarinic acid in HPLC‐UV analysis, alongside commonly spread hydroxycinnamic acids (Basaraba et al. 2022). Owing to the fact that chlorogenic acid together with quercetin glycosides were commonly found in Asteraceae, the chemophenetic significance of these secondary metabolites could not be discussed. On the other hand, acylhexaric (glucaric) acids have been delineated as chemophenetic markers of the tribe Gnaphalieae with data strongly suggesting that the *Gnaphalium* L. and *Leontopodium* (Pers.) R.Br. ex Cass. species are sources of leontopodic acids A and B (Cicek et al. 2012). Scare data have been reported on the antioxidant potential of *A. dioica* flowering heads: the most prominent activity has been evaluated for the ethanol‐aqueous extract in 2,2‐DiPhenyl‐1‐PicrylHydrazyl (DPPH) (15.21 ± 1.97 mg TE/mL) and Trolox Equivalent Antioxidant Capacity (TEAC) (5.71 ± 0.01 mg TE/mL) assays (Babotă et al. 2018).

It is important to note that there is no in‐depth metabolic profile of *A. dioica* using liquid chromatography coupled to high resolution mass spectrometry (LC‐HRMS). Our goal was to investigate whether the *A. dioica* aerial parts contain acylhexaric acids alongside acylquinic acids (AQAs) and flavonoids (notably methoxylated derivatives) and whether the 80% methanol extract exhibit promising activity in the antioxidant assays by different mechanisms. Beyond antioxidant capacity, phenolics in Asteraceae are frequently associated with enzyme inhibition relevant to metabolic and neurodegenerative targets (e.g., *α*‐amylase/*α*‐glucosidase, cholinesterases, tyrosinase) (Liao et al. 2025; Tadera et al. 2006; Zengin et al. 2022). In general, the cited investigations generate additional interest in the *A. dioica* and prompted us to evaluated the enzyme inhibitory potential towards pharmacology relevant targets in therapies.

LC‐HRMS enables broad, sensitive annotation of specialized metabolites and can be integrated with in vitro bioassays and in silico approaches to help rationalize SAR (structure–activity relationship) at enzyme targets (Koulis et al. 2021; Quiros‐Guerrero et al. 2024; Zengin et al. 2022). Although numerous studies have reported docking investigations of AChE and BChE (Gok et al. 2025; http://www.tandfonline.com/loi/gsar20, n.d.; Khedraoui et al. 2024; Nguyen and Kim 2024; Pang et al. 2017), the continued search for effective therapies for neurodegenerative diseases underscores the importance of identifying new candidate molecules, particularly those of natural origin. Likewise, several publications describe in silico assessments of plant‐derived compounds as *α*‐glucosidase and *α*‐amylase inhibitors in the search for novel agents to manage metabolic disorders, indicating that additional screening for promising ligands remains justified (Aispuro‐Pérez et al. 2020; Liu et al. 2020; Rahman et al. 2019). In this study, to understand the mechanisms of the inhibitory activity of plant extract, we performed docking to AChE (PDB code 4EY7), BChE (PDB code 7AMZ), *α*‐amylase (PDB code 1Z32), and *α*‐glucosidase (PDB code 5NN6). By bridging phytochemical annotation with bioactivity and modeling, we aim to clarify the molecular basis of the plant's traditional uses and to identify hit molecules worthy of further pharmacological evaluation.

Applying this approach to *A. dioica*, our study provides a route to (i) comprehensive UHPLC‐HRMS/MS phytochemical profiling; (ii) correlate metabolite features with antioxidant and enzyme‐inhibitory activities; and (iii) propose mechanistic hypotheses for key constituents via molecular modeling using ADAME/Tox.

An innovative workflow based on the association of UHPLC‐HRMS/MS with pharmacological activity and in silico investigation to target bioactive compounds from the extract was developed. In addition, the significance of some secondary metabolites found for the first time in *A. dioica* was delineated as chemophenetic markers of the tribe Gnaphalieae. The potential of *A. dioica* as a rich source of caffeoylhexaric, including leontopodic acids A and B, as well as its beneficial effects on the metabolic disorders and skin imperfections, and oxidative stress was discussed for the first time.

## 2 Materials and Methods

### 2.1 Plant Material

*Antennaria dioica* aerial parts were collected at “Cherni vrah,” Vitosha Mt. (2280 m. a.s.l.), Bulgaria, during the flowering stage in July 2023. The plant species was identified by one of us (D.Z.) according to World Flora Online (https://www.worldfloraonline.org, n.d.). A voucher specimen was deposited at the Herbarium of the Institute of Biodiversity and Ecosystem Research, Bulgarian Academy of Sciences (SOM) (No. 179540). The collected plant material was dried at room temperature.

### 2.2 Sample Extraction

*A. dioica* aerial parts were ground (Rohnson grinder, R‐942, 220–240 V, 50/60 Hz, 200 W, Prague, Czech Republic). Then, 10 g was extracted using 80% MeOH (1:20 w/v) and ultrasound (100 kHz, ultra‐sound bath Biobase UC‐20C, Shandong, China) two times for 15 min at room temperature. Then, methanol was evaporated in vacuo (at 40°C) and the water residue was lyophilized (Biobase BK‐FD10P, Shandong, China; −65°C). 2.1 g of crude extract was obtained. The crude lyophilized extract was dissolved in 80% methanol (0.1 mg/mL), filtered through a 0.45 μm syringe filter, and an aliquot (2 mL) was subjected to further UHPLC–HRMS/MS analyses. The same extract was used for the antioxidant and enzyme inhibitory tests.

### 2.3 Chemicals

Acetonitrile (for LC–MS), formic acid (for LC–MS), and methanol (for HPLC) were provided from Chromasolv (Sofia, Bulgaria). The reference standards were provided by Extrasynthese (Genay, France) (for leontopodic acid A, leontopodic acid B, protocatechuic, gentisic, caffeic, vanillic, *p*‐, m‐, *o*‐coumaric acids, rutin, kaempferol 3‐*O*‐rutinoside, apigenin 7‐*O*‐glucoside, luteolin 7‐*O*‐glucoside, isorhamnetin 3‐*O*‐rutinoside, apigenin, luteolin, kaempferol, quercetin, isorhamnetin, and genkwanin) and Phytolab (Vesten‐bergsgreuth, Bavaria, Germany) (chlorogenic, neochlorogenic acids, 1,5‐di‐, 3,4‐di‐, and 4,5‐dicaffeoylquinic acids, nepetin 7‐*O*‐glucoside, nepetin, and scopoletin). A working solution (0.1 mg/mL) of the tested compounds was prepared from a stock solution in MeOH (0.5 mg/mL).

The chemicals used for the determination of antioxidant and enzyme inhibition tests ABTS, DPPH, 2,4,6‐Tris (2‐pyridyl)‐s‐triazine (TPTZ), acetylcholinesterase from electric eel (AChE) (type‐VI‐S, EC 3.1.1.7), butyrylcholinesterase from horse serum (BChE) (EC 3.1.1.8), acetylthiocholine iodide (ATChI), butyrylthiocholine iodide (BTChI), amylase (EC. 3.2.1.1, from porcine pancreas), glucosidase (EC. 3.2.1.20, from *Saccharomyces cerevisiae* ), tyrosinase (EC1.14.18.1, mushroom), galantamine, gallic acid, rutin, Folin–Ciocalteu reagent, sodium molybdate, hydrochloric acid, sodium hydroxide, sodium carbonate, trolox, ethylenediaminetetraacetate (EDTA), cupric chloride, neocuproine, ammonium acetate, ferric chloride, 5,5‐dithio‐bis (2‐nitrobenzoic) acid (DTNB), acarbose, ammonium molybdate, ferrozine, ferrous sulfate hexahydrate, and kojic acid and were purchased from Sigma‐Aldrich (Darmstadt, Germany).

### 2.4 UHPLC‐HRMS/MS

The UHPLC‐HRMS/MS analyses were carried out as previously described (Gevrenova et al. 2020) on a Q Exactive Plus mass spectrometer (ThermoFisher Scientific Inc., Waltham, MA, USA), in negative ion mode with the *m/z* range from 150 to 1500, at a resolution of 70,000. Other instrument parameters for Full MS mode were set as follows: automatic gain control (AGC) target 3e6, maximum injection time (IT) 100 ms, number of scan ranges 1. For the DD‐MS2 mode, the instrument parameters were as follows: microscans 1, resolution 17,500, AGC target 1e5, maximum IT 50 ms, MSX count 1, Top5, isolation window 2.0 m/z, stepped normalized collision energy (NCE) 10, 20, 60 eV. The separation was performed on a C18 column and the temperature was 40°C. The mobile phase contained 0.1% formic acid in water (A) and 0.1% formic acid in acetonitrile (B). The gradient elution program was as follows: 0–1 min from 0% to 5% B, 20 min—30% B, 25 min—50% B, 30 min—70% B, 33 min—95%. The flow rate was 0.3 mL/min, the injection volume was 1 μL. Data acquisition was processed by software Xcalibur 4.2 (ThermoScientific). MZmine 2 software was used to process the UHPLC–HRMS raw files for the further semi‐quantitative analysis. The peak areas were calculated by integrating the Area Under the Curve (AUC) of the full‐scan intensity scans for the corresponding molecular ion. These scans were also filtered for the presence of the characteristic base peak. Results are expressed as the percentage peak area of each compound to the total peak areas of the group secondary metabolites (Gevrenova et al. 2020).

### 2.5 Assay for Total Phenolic and Flavonoid Contents

Total phenolics and flavonoids in the studied extract were quantified according to the methods (Folin–Ciocalteu assay for total phenolic content and AlCl_3_ method for total flavonoid content) specified by Zengin and Aktumsek (2014). Gallic acid and rutin were used as positive controls, and the results were expressed as gallic acid equivalents (GAEs) and rutin equivalents (REs).

### 2.6 Determination of Antioxidant and Enzyme Inhibitory Activity

The determination of antioxidant and enzyme inhibitory activity of the tested extract was performed according to Zengin et al. (2016). The used extract concentrations ranged from 0.1 to 2 mg/mL. The results for the DPPH, ABTS radical scavenging, CUPRAC, and FRAP experiments were presented as milligrams of Trolox equivalents per gram of dry extract (mgTE/g). The antioxidant activity determined by the phosphomolybdenum (PBD) method was expressed as millimoles of Trolox equivalents per gram of dry extract (mMTE/g). Metal chelating activity (MCA) was presented as milligrams of disodium edetate equivalents per gram of dry extract (mg EDTAE/g). Acetylcholinesterase (AChE) and butyrylcholinesterase (BChE) inhibition was presented as milligrams of galanthamine equivalents per gram of extract (mg GALAE/g), while amylase and glucosidase inhibitory potential was expressed as acarbose equivalents per gram of dry extract (mgACAE/g). Lipase inhibitory activity was measured as equivalents of orlistat per gram of dry extract (mgOE/g). Tyrosinase inhibitory activity was evaluated in milligrams of kojic acid equivalents per gram of dry extract (mgKAE/g).

### 2.7 Statistical Analysis

All experiments were performed in triplicate, and the results are expressed as mean ± standard deviation (SD). Since only a single extract was analyzed, no inferential statistical tests or significance thresholds were applied. The data were evaluated using descriptive statistical methods only. GraphPad 9.1 software was used to calculate the obtained results (Zengin et al. 2016).

### 2.8 In Slilico Investigation

#### 2.8.1 Preparation of Ligand Structures

The SMILES of the investigated chemical compounds were taken from PubChem or were written manually. All positional isomers were investigated for compounds with only one isomer found in the extract but not completely defined. For compounds with unknown cis/trans isomerism, trans structures were selected (as being more stable in general) except in the cases with more isomers and no other cis state possibility was found. *β*‐glucose and glucaric acid were used in the cases where the hexose and hexaric acid, respectively, were not known, due to being more widespread and having higher stability.

The chemical structures were built with Molecular Operating Environment (MOE) software v. 2024.0601 (Chemical Computing Group ULC, 910‐1010 Sherbrooke St. W., Montreal, QC H3A 2R7, 2025) using the derived SMILES. Before docking the compounds, energies were minimized using the AmberEHT force field.

#### 2.8.2 Protein Structures Selection

All crystallographic structures of human AChE, BChE, *α*‐amylase and *α*‐glucosidase available in the Protein Data Bank (PDB) (Berman 2000; http://www.rcsb.org, n.d.) were analyzed in order to choose the protein structures for the in silico investigation. Protein‐ligand complexes with better X‐ray resolution and a smaller number of unresolved residues were selected. Thus, the following complexes were chosen: 4EY7 for AChE (in complex with donepezil (E20); resolution 2.35 Å, protein B chain was used in the docking) (Cheung et al. 2012), 7AMZ for BChE (complex with 2,2‐dimethylpropyl‐[(2~{R},3~{S})‐3‐(2,2‐diphenylethanoylamino)‐2‐oxidanyl‐4‐phenyl‐butyl]azanium (RNZ), 2.25 Å resolution) (Pasieka et al. 2021), 1Z32 for *α*‐amylase (with a ligand composed of alpha‐D‐glucopyranose (GLG), 5‐hydroxymetyl‐chonduritol (HMC), and 4‐amino‐4,6‐dideoxy‐alpha‐D‐glucopyranose (ALG) covalently bound, referred altogether as GLC_AGL_HMC in the paper, including Ca^2+^ ion, 1.60 Å resolution) (Ramasubbu et al. 2005), and 5NN6 for *α*‐glucosidase (in complex with (2R,3R,4R,5S)‐1‐(2‐hydroxyethyl)‐2‐(hydroxymethyl)piperidine‐3,4,5‐triol (MIC), 2.0 Å resolution) (Roig‐Zamboni et al. 2017). In all complexes the crystallographic water molecules were kept in the investigation.

Human tyrosinase and human lipase were excluded from the study because no crystallographic structures of these enzymes in non‐covalent complexes with ligands were found in the PDB.

#### 2.8.3 Protein Structures Preparation

Protein structures were prepared using the “Protonate 3D” tool in MOE, which optimizes the protein by (i) adding hydrogens according to the lowest freeenergy proton geometry and (ii) assigning ionization states of titratable residues based on the Generalized Born electrostatics model. To mimic physiological conditions, the process was performed at 310 K, pH 7.4, and an ion concentration of 0.152 mol/L.

#### 2.8.4 Docking Protocol

Docking was done with MOE v. 2024.0601, using triangle matcher placement method. The protein binding sites were defined based on the crystallographic ligand atoms. Poses with lower negative docking scores (having higher absolute values) are considered to have better binding to the enzyme. The 10 poses with the best (lowest) docking scores were kept.

In order to validate the docking protocol re‐docking of the crystallographic ligands was done using different combinations of scoring functions for the placement and the refinement of the poses, including London dG, GBVI/WSA dG, and Alpha HB (Kalinowsky et al. 2018) functions. The best root mean square deviation (RMSD) results between the crystallographic ligand and the re‐docked poses were obtained with the combination of London dG for the placement and GBVI/WSA dG for the refinement of the poses (data not shown); therefore, they were used for the subsequent docking of the investigated compounds.

Visualization of the protein‐ligand interactions was done with MOE v. 2024.0601, “Compute Ligand Interactions” tool.

#### 2.8.5 ADME/Tox Calculations

ACD/Percepta software (ACD/Labs Release 2024.2.3, Advanced Chemistry Development Inc., Toronto, ON, Canada, https://www.acdlabs.com) and Derek Nexus v.6.4.2. expert system (Lhasa Limited, Leeds, UK, https://www.lhasalimited.org) were used to calculate ADME/Tox properties of the investigated compounds.

ACD/Percepta software was used to calculate molecular weight (MW), log*p* (partition coefficient oil/water), human intestinal absorption (HIA), probability for positive Ames test, hERG Inhibitor (Ki < 10 μM) probability, blood–brain penetration, and probability for substrates and inhibitors of Pgp. The Reliability Index (RI) provided in ACD/Percepta calculations indicates the confidence level of the prediction. An RI value below 0.3 suggests that the compound structure is different from the training data used to build the prediction model, and the prediction is unreliable.

The predictions with Derek Nexus were done for mammal species. Derek Nexus generates a prediction by comparing the structural features of the compound with a toxicophore (structural alert) encoded as structural pattern(s) in its knowledge base. The final predictions are derived from a reasoning scheme which takes into account other relevant factors, for example physicochemical properties, and presence of a toxicophore in the query structure [https://doi.org/10.1080/15376510701857320]. The predictions are provided with the following levels of likelihood, from highest to lowest, respectively: “certain,” “probable,” “plausible,” “equivocal,” “doubted,” “improbable,” and “impossible” (Judson et al. 2013). In this study, the level of likelihood “plausible” was selected as a threshold, meaning “the weight of evidence supports the proposition.”

## 3 Results and Discussion

**FIGURE 1:**
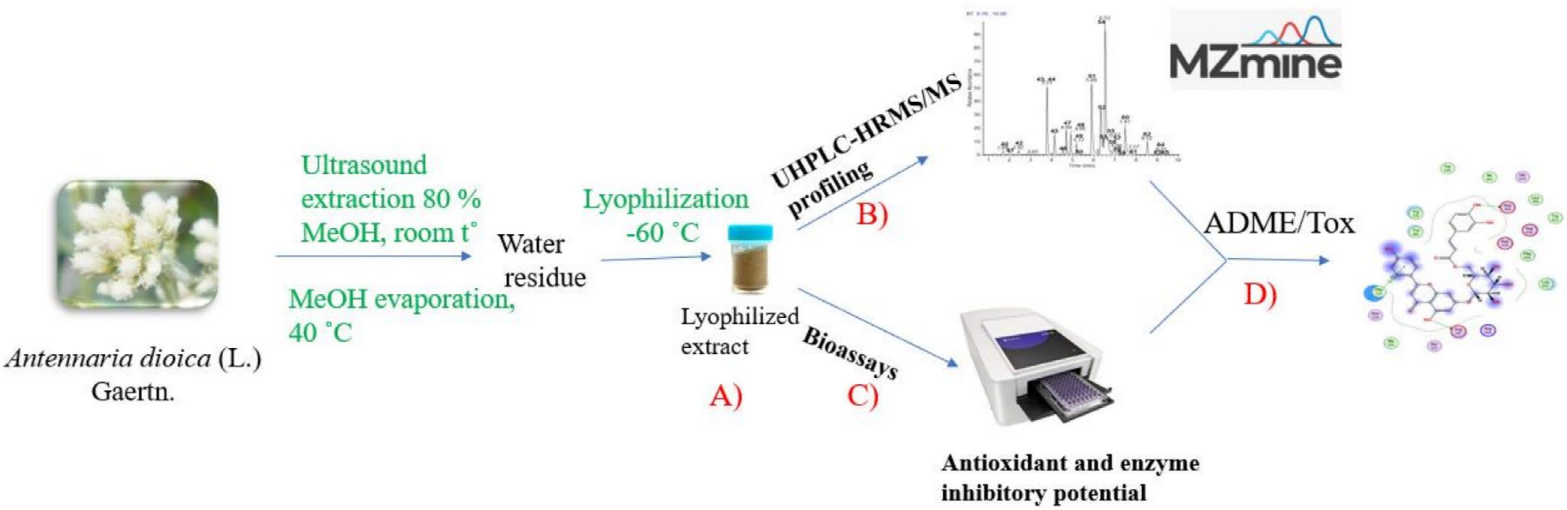
Flowchart for the *Antennaria dioica* study, including plant extraction procedure (A); UHPLC‐HRMS/MS profiling where data are acquired using the data‐dependent acquisition mode, then converted through a MZmine 2 software processing (B). In parallel, spectrophotometric bioassays (C) are conducted to determine antioxidant (DPPH, ABTS, CUPRAC, FRAP, PBD, metal chelating) and enzyme inhibitory activity (against AChe, BChE, ɑ‐amylase, ɑ‐glucosidase, lipase, tyrosinase) and information are tabulated. The final step is to correlate metabolite features with antioxidant and enzyme‐inhibitory activities; and propose mechanistic hypotheses for key constituents via molecular modeling using ADAME/Tox (D).

A flowchart combining plant extraction procedure, UHPLC‐HRMS/MS data annotation, evaluation of the extract biological potential and in silico investigation is presented on Figure 1.

### 3.1 UHPLC‐HRMS/MS Profiling of *A. dioica* Extract

The UHPLC‐HRMS/MS analysis of *A. dioica* methanol‐aqueous extract was performed allowing for the identification/annotation of 130 secondary metabolites (Table 1). Based on the MS accurate masses, MS/MS fragmentation patterns, relative ion abundances, retention times, and comparison with reference standards or/and literature data, 39 acylquinic, 26 caffeoylhexaric, 34 carboxylic, phenolic acids and coumarins, as well as 31 flavonoids were annotated in the extract. Their retention times, molecular formulas, MS/MS fragment ions, and mass measurement errors are depicted in Table 1. Identification confidence levels for metabolite profiling were previously described and were as follows: A2: confirmed structure including confirmed stereochemistry; B: confirmed structure except for one or more stereochemical aspects; C: tentative identification matched with a standard compound, match of at least *t*_R_, MS and MS/MS with an actual authentic standard analyzed in parallel, preferably supported by other online data; D: tentative identification based on libraries, model compounds etc.; D1: relatively reliable evidence; D2: relatively poor evidence (Çiçek et al. 2024).

**TABLE 1:** Secondary metabolites in *Antennaria dioica* methanol‐aqueous extract.

№	Identified/tentatively annotated compound	Molecular formula/[2 M‐H]^−^	Exact mass [M‐H]^−^	Fragmentation pattern in (−) ESI‐MS/MS [M‐H]^−^ and [2 M‐H]^−^ in EIC	*t*_R_ (min)	Δ ppm	Confidence level	References
**Acylquinic acids**								
1.	1‐hydroxy‐dihydrocaffeoylquinic acid^a^	C_16_H_20_O_10_ C_32_H_39_O_20_	371.0984	371.0983 (24.2), 197.0448 (1.8), 191.0552 (10.3), 179.0337 (5.7), 173.0444 (7.9), 161.0231 (0.3), 135.0437 (100), 111.0436 (0.8), 93.0330 (1.9), 85.0279 (1.0) [M‐H]^−^ 371.0992 (35.6)	1.08	−0.108	D2	Chawech et al. (2022)
2.	5‐ hydroxy‐dihydrocaffeoylquinic acid^a^	C_16_H_20_O_10_ C_32_H_39_O_20_	371.0984	371.0984 (34.2), 197.0446 (1.3), 191.0551 (100), 173.0444 (14.6), 161.0230 (3.3), 135.0437 (11.8), 111.0434 (1.2), 93.0330 (7.5), 85.0279 (7.6) [M‐H]^−^ 371.0989 (17.7), [2 M‐H]^−^ 743.2073 (0.02)	1.26	0.054	D2	Chawech et al. (2022)
3.	4‐ hydroxy‐dihydrocaffeoylquinic acid^a^	C_16_H_20_O_10_ C_32_H_39_O_20_	371.0984	371.0984 (70.9), 353.0878 (12.8), 197.0445 (3.8), 191.0551 (98.1), 179.0338 (10.3), 173.0444 (100), 135.0437 (56.5), 111.0438 (5.1), 93.0330 (5.1), 85.0278 (14.7) [M‐H]^−^ 371.0992 (45.9), [2 M‐H]^−^ 743.2085 (0.1)	1.40	0.054	D2	Chawech et al. (2022)
4.	Neochlorogenic acid^a^, ^b^	C_16_H_18_O_9_ C_32_H_35_O_18_	353.0867	353.0879 (45.3), 191.0551 (100), 179.0339 (59.5), 173.0446 (3.4), 161.0233 (3.8), 135.0436 (52.6), 111.0434 (2.2), 93.0329 (4.9), 85.0279 (9.8) [M‐H]^−^ 353.0879 (100), [2 M‐H]^−^ 707.1834 (12.5)	2.37	0.155	A2	Clifford et al. (2003)
5.	4‐caffeoylquinic acid 1^a^	C_16_H_18_O_9_ C_32_H_35_O_18_	353.0867	353.0879 (48.6), 191.0550 (54.7), 179.0339 (68.7), 173.0443 (100), 161.0226 (3.1), 135.0437 (56.4), 111.0436 (3.6), 93.0330 (22.5), 85.0279 (9.5) [M‐H]^−^ 353.0883 (46.5), [2 M‐H]^−^ 707.1853 (5.8)	3.05	0.240	D1	Clifford et al. (2003)
6.	3‐*p*‐coumaroylquinic acid^a^	C_16_H_18_O_8_ C_32_H_35_O_16_	337.0928	337.0931 (12.8), 191.0552 (32.2), 173.0444 (4.5), 163.0388 (100), 119.0487 (31.6), 111.0431 (0.3), 93.0330 (2.5), 85.0277 (2.9) [M‐H]^−^ 337.0937 (59.9), [2 M‐H]^−^ 675.1954 (0.3)	3.05	0.562	D1	Clifford et al. (2003)
7.	Chlorogenic acid^b^	C_16_H_18_O_9_ C_32_H_35_O_18_	353.0867	353.0878 (4.9), 191.0551 (100), 179.0333 (1.0), 173.0442 (0.8), 161.0231 (1.7), 135.0438 (1.9), 111.0433 (0.9), 93.0329 (3.1), 85.0279 (8.6) [M‐H]^−^ 353.0880 (100), [2 M‐H]^−^ 707.1832 (74.9)	3.19	0.400	A2	Clifford et al. (2003)
8.	Dihydrocaffeoylquinic acid^a^	C_16_H_20_O_9_ C_32_H_39_O_18_	355.1035	355.1030 (16.7), 191.0550 (100), 173.0443 (5.3), 137.0589 (0.4), 127.0386 (2.0), 111.0436 (1.8), 93.0330 (11.8), 85.0279 (12.5) [M‐H]^−^ 355.0933 (2.7)	3.20	−1.367	D2	Clifford et al. (2003)
9.	4‐caffeoylquinic acid 2^a^	C_16_H_18_O_9_ C_32_H_35_O_18_	353.0867	353.0879 (35.8), 191.0551 (51.4), 179.0339 (62.4), 173.0444 (100), 161.0232 (2.3), 135.0437 (51.4), 111.0435 (51.4), 111.0435 (3.6), 93.0330 (22.7), 85.0279 (8.6) [M‐H]^−^ 353.0880 (100), [2 M‐H]^−^ 707.1831 (18.5)	3.37	0.325	D1	Clifford et al. (2003)
10.	3‐feruloylquinic acid^a^	C_17_H_20_O_8_ C_34_H_39_O_16_	367.1034	367.1036 (20.4), 193.0497 (100), 191.0553 (2.5), 173.0445 (4.7), 149.0595 (3.8), 134.0359 (57.3), 111.0438 (1.2), 93.0330 (1.7) [M‐H]^−^ 367.1039 (100)	3.45	0.503	D1	Clifford et al. (2003)
11.	5‐caffeoylquinic acid isomer^a^	C_16_H_18_O_9_ C_32_H_35_O_18_	353.0867	353.0877 (5.2), 191.0550 (100), 179.0340 (0.7), 173.0441 (0.9), 161.0230 (2.2), 135.0435 (0.3), 111.0434 (1.1), 93.0331 (2.5), 85.0279 (8.6) [M‐H]^−^ 353.0879 (100), [2 M‐H]^−^ 707.1835 (16.6)	3.90	−0.270	D1	Clifford et al. (2003)
12.	5‐*p*‐coumaroylquinic acid 1^a^	C_16_H_18_O_8_ C_32_H_35_O_16_	337.0928	337.0927 (8.7), 191.0550 (100), 173.0443 (5.8), 163.0387 (6.5), 119.0487 (5.0), 111.0437 (2.6), 93.0330 (18.2), 85.0279 (4.6) [M‐H]^−^ 337.0934 (100), [2 M‐H]^−^ 675.1953 (3.0)	3.96	0.384	D1	Clifford et al. (2003)
13.	3‐caffeoyl‐5‐hydroxy‐dihydrocaffeoylquinic acid^a^	C_25_H_26_O_13_	533.1288	533.1312 (63.3), 371.0613 (17.2), 353.0880 (17.6), 335.0784 (4.0), 191.0550 (100), 179.0338 (41.1), 173.0443 (15.2), 161.0228 (6.8), 135.0436 (57.2), 111.0436 (3.0), 93.0329 (8.7), 85.0278 (17.6)	4.03	2.056	D2	Ren et al. (2018)
14.	3‐caffeoyl‐4‐ hydroxy‐dihydrocaffeoylquinic acid^a^	C_25_H_26_O_13_	533.1288	533.1307 (100), 371.0998 (8.7), 353.0878 (6.1), 335.0776 (10.5), 191.0552 (9.0), 179.0339 (17.4), 173.0443 (85.7), 161.0231 (14.6), 135.0436 (63.2), 111.0435 (4.6), 93.0329 (19.2); 85.0279 (5.7)	4.34	1.268	D2	Ren et al. (2018)
15.	5‐feruloylquinic acid 1^a^	C_17_H_20_O_8_ C_34_H_39_O_16_	367.1034	367.1032 (15.8), 193.0497 (4.6), 191.0550 (100), 173.0443 (11.5), 149.0591 (0.2), 134.0359 (9.1), 111.0434 (3.0), 93.0329 (23.6), 85.0278 (4.5) [M‐H]^−^ 367.1037 (100), [2 M‐H]^−^ 735.2164 (8.8)	4.41	−0.668	D1	Clifford et al. (2003)
16.	1‐caffeoyl‐3‐hydroxydihydrocaffeoylquinic acid^a^	C_25_H_26_O_13_	533.1288	533.1307 (29.1), 371.0985 (53.3), 353.0880 (5.5), 335.0771 (2.7), 191.0551 (14.3), 179.0340 (12.7), 173.0444 (27.5), 161.0231 (4.9), 135.0437 (100), 93.0330 (8.3), 85.0277 (2.3) [M‐H]^−^ 533.1306 (67.2)	4.43	1.268	D2	Ren et al. (2018)
17.	5‐*p*‐coumaroylquinic acid 2^a^	C_16_H_18_O_8_ C_32_H_35_O_16_	337.0928	337.0930 (5.7), 191.0549 (100), 173.0444 (1.9), 163.0387 (1.9), 119.0488 (1.2), 111.0435 (1.2), 93.0329 (5.0), 85.0278 (7.4) [M‐H]^−^ 337.0930 (100), [2 M‐H]^−^ 675.1935 (20.0)	4.62	0.206	D1	Clifford et al. (2003)
18.	4‐feruloylquinic acid^a^	C_17_H_20_O_8_ C_34_H_39_O_16_	367.1034	367.1034 (100), 193.0496 (11.6), 191.0554 (3.1), 173.0443 (69.3), 163.0388 (15.9), 149.0597 (0.5), 134.0359 (23.4), 111.0435 (13.6), 93.0329 (99.2), 85.0278 (0.6) [M‐H]^−^ 367.1… (100), [2 M‐H]^−^ 735.2141 (8.8)	4.67	−0.178	D1	Clifford et al. (2003)
19.	5‐feruloylquinic acid 2^a^	C_17_H_20_O_8_ C_34_H_39_O_16_	367.1034	367.1031 (15.3), 193.0497 (1.5), 191.0549 (100), 179.0337 (3.5), 173.0447 (2.2), 161.0229 (1.3), 134.0359 (2.7), 111.0437 (1.1), 93.0329 (5.4), 85.0278 (7.6) [M‐H]^−^ 367.1038 (32.1), [2 M‐H]^−^ 735.2170 (0.6)	4.92	0.954	D1	Clifford et al. (2003)
20.	1, 4‐dicaffeoylquinic acid^a^	C_25_H_24_O_12_ C_50_H_47_O_24_	515.1189	515.1196 (100), 353.0879 (18.2), 335.0771 (6.7), 191.0551 (32.4), 179.0338 (49.0), 173.0443 (60.9), 161.0231 (18.2), 135.0437 (49.6), 111.0434 (3.7), 93.0329 (11.1), 85.0279 (3.9) [M‐H]^−^ 515.1196 (43.3), [2 M‐H]^−^ 1031.2498 (0.03)	5.55	0.254	D1	Clifford et al. (2005)
21.	3, 4‐dicaffeoylquinic acid^a^, ^b^	C_25_H_24_O_12_ C_50_H_47_O_24_	515.1189	515.1196 (100), 353.0879 (10.5), 335.0769 (6.4), 203.0352 (0.5), 191.0551 (18.2), 179.0339 (31.2), 173.0443 (37.7), 161.0230 (10.9), 135.0436 (31.7), 111.0434 (2.7), 93.0328 (10.7), 85.0278 (1.5) [M‐H]^−^ 515.1191 (100), [2 M‐H]^−^ 1031.2482 (9.9)	5.68	0.196	A2	Clifford et al. (2005)
22.	3,5‐dicaffeoylquinic acid^a^	C_25_H_24_O_12_ C_50_H_47_O_24_	515.1189	515.1194 (16.1), 353.0878 (100), 335.0798 (0.6), 191.0550 (84.5), 179.0338 (42.0), 161.0229 (3.9), 135.0437 (45.1), 93.0327 (3.6), 85.0278 (6.6) [M‐H]^−^ 515.1191 (100), [2 M‐H]^−^ 1031.2462 (16.8)	5.84	0.972	C	Clifford et al. (2005)
23.	1,5‐dicaffeoylquinic acid^a^, ^b^	C_25_H_24_O_12_ C_50_H_47_O_24_	515.1189	515.1198 (33.4), 353.0879 (95.4), 335.0768 (2.2), 191.0551 (100), 179.0339 (58.5), 173.0443 (10.7), 161.0231 (6.1), 135.0437 (46.6), 111.0434 (1.6), 93.0331 (4.6), 85.0279 (6.7) [M‐H]^−^ 515.1193 (100), [2 M‐H]^−^ 1031.2490 (8.9)	6.03	0.487	A2	Clifford et al. (2005)
24.	4,5‐dicaffeoylquinic acid 1^a^	C_25_H_24_O_12_ C_50_H_47_O_24_	515.1189	515.1197 (99.6), 353.0877 (64.7), 335.0776 (0.6), 191.0551 (34.8), 179.0339 (65.5), 173.0444 (100), 135.0437 (64.6), 111.0436 (3.8), 93.0330 (23.4), 85.0279 (5.2) [M‐H]^−^ 515.1190 (100), [2 M‐H]^−^ 1031.2478 (9.6)	6.23	0.370	C	Clifford et al. (2005)
25.	1‐*p*‐coumaroyl‐4‐caffeoylquinic acid^a^	C_25_H_24_O_11_ C_50_H_47_O_22_	499.1251	499.1250 (100), 353.0878 (8.5), 337.0927 (7.1), 335.0775 (9.4), 319.0822 (5.3), 191.0551 (10.1), 179.0339 (33.6), 173.0443 (43.1), 163.0387 (48.2), 161.0230 (16.1), 145.0281 (9.5), 135.0436 (34.4), 119.0486 (17.7), 111.0434 (4.7), 93.0329 (12.2), 85.0279 (2.0) [M‐H]^−^ 499.1259 (2.6) [2 M‐H]^−^ nd	6.32	0.892	D1	Clifford et al. (2003)
26.	3‐caffeoyl‐4‐ *p*‐coumaroylquinic acid^a^	C_25_H_24_O_11_ C_50_H_47_O_22_	499.1251	499.1247 (73.6), 337.0929 (20.9), 335.0770 (3.7), 319.0834 (1.3), 191.0555 (2.1), 179.0337 (4.8), 173.0443 (100), 163.0388 (16.6), 161.0231 (9.9), 145.0275 (2.7), 135.0437 (7.9), 127.0384 (1.4), 119.0487 (8.7), 111.0434 (3.1), 93.0329 (20.3), 85.0279 (0.4) [M‐H]^−^ 499.1253 (15.3), [2 M‐H]^−^ 999.2598 (0.1)	6.43	0.291	D1	Ren et al. (2018)
27.	3‐feruloyl‐4‐caffeoylquinic acid^a^	C_26_H_26_O_12_ C_52_H_51_O_24_	529.1356	529.1351 (17.4), 367.1035 (6.5), 353.0878 (6.9), 349.0931 (6.7), 335.0776 (10.4), 193.0497 (51.4), 179.0339 (32.1), 173.0444 (38.1), 161.0232 (19.2), 149.0594 (1.4), 135.0439 (33.9), 134.0359 (39.2), 111.0436 (5.4), 93.0330 (10.8) [M‐H]^−^ 529.1363 (10.4), [2 M‐H]^−^ 1059.2817 (0.1)	6.50	0.776	D1	Clifford et al. (2003)
28.	3‐*p*‐coumaroyl‐5‐caffeylquinic acid^a^	C_25_H_24_O_11_ C_50_H_47_O_22_	499.1251	499.1250 (20.0), 353.0852 (0.9), 337.0930 (76.4), 191.0551 (10.5), 179.0334 (0.9), 173.0444 (9.4), 163.0387 (100), 135.0437 (3.0), 119.0487 (38.9), 111.0436 (1.9), 93.0329 (4.0), 85.0277 (0.7) [M‐H]^−^ 499.1255 (2.5) [2 M‐H]^−^ nd	6.52	0772	D1	Clifford et al. (2003)
29.	3‐caffeyl‐5‐*p*‐coumaroylquinic acid 1^a^	C_25_H_24_O_11_ C_50_H_47_O_22_	499.1251	499.1250 (30.1), 353.0879 (67.1), 337.0931 (27.1), 319.0815 (1.3), 191.0551 (100), 179.0339 (38.2), 173.0445 (0.3), 163.0388 (10.6), 145.0280 (2.7), 135.0437 (41.4), 119.0487 (6.8), 111.0437 (2.4), 93.0330 (12.4), 85.0278 (6.7) [M‐H]^−^ 499.1252 (16.4), [2 M‐H]^−^ 999.2614 (0.1)	6.58	0.832	D1	Clifford et al. (2003), Ren et al. (2018)
30.	3‐caffeoyl‐4‐feruloylquinic acid^a^	C_26_H_26_O_12_ C_52_H_51_O_24_	529.1356	529.1352 (21.4), 367.1031 (18.5), 349.0930 (1.3), 335.0775 (3.6), 193.0494 (18.9), 179.0336 (6.3), 173.0443 (100), 161.0231 (10.9), 149.0593 (0.5), 135.0439 (9.0), 134.0359 (16.9), 111.0435 (4.6), 93.0329 (23.4), 85.0277 (1.3) [M‐H]^−^ 529.1364 (4.6), [2 M‐H]^−^ nd	6.63	0.077	D1	
31.	3‐caffeoyl‐5‐*p*‐coumaroylquinic acid 2/1‐*p*‐coumaroyl‐5‐caffeylquinic acid^a^	C_25_H_24_O_11_ C_50_H_47_O_22_	499.1251	499.1248 (47.2), 353.0879 (51.6), 337.0931 (32.9), 335.0785 (1.3), 319.0826 (1.2), 191.0551 (100), 179.0339 (38.2), 173.0444 (28.8), 163.0388 (15.9), 161.0231 (7.4), 145.0279 (2.3), 135.0437 (41.7), 119.0488 (8.7), 111.0434 (3.1), 93.0329 (11.8), 85.0279 (7.7) [M‐H]^−^ 499.1251 (100), [2 M‐H]^−^ 999.2603 (3.2)	6.78	0.471	D1	Clifford et al. (2003), Ren et al. (2018)
32.	3‐feruloyl‐5‐caffeoylquinic acid^a^	C_26_H_26_O_12_ C_52_H_51_O_24_	529.1356	529.1351 (15.8), 367.1033 (33.8), 335.0770 (1.2), 193.0496 (100), 191.0552 (8.6), 179.0337 (1.4), 173.0444 (9.8), 161.0232 (8.3), 135.0400 (37.6), 134.0358 (67.9), 111.0434 (1.8), 93.0331 (4.2), 85.0278 (1.9) [M‐H]^−^ 529.1360 (19.8), [2 M‐H]^−^ 1059.2826 (0.1)	6.82	0.776	D1	
33.	3‐caffeoyl‐5‐feruloylquinic acid^a^	C_26_H_26_O_12_ C_52_H_51_O_24_	529.1356	529.1362 (12.8), 367.1036 (30.6), 353.0879 (39.6), 335.0757 (0.6), 191.0551 (100), 179.0339 (37.6), 173.0444 (10.9), 161.0232 (6.5), 149.0595 (0.4), 135.0438 (37.8), 134.0359 (16.3), 111.0437 (3.4), 93.0330 (14.7), 85.0279 (7.9) [M‐H]^−^ 529.1358 (32.1), [2 M‐H]^−^ 1059.2839 (0.1)	6.88	1.929	D1	Clifford et al. (2003), Ren et al. (2018)
34.	4‐*p*‐coumaroyl‐5‐caffeoylquinic acid^a^	C_25_H_24_O_11_ C_50_H_47_O_22_	499.1251	499.1245 (23.4), 353.0874 (2.4), 337.0930 (68.1), 191.0551 (8.4), 179.0339 (2.5), 179.0339 (2.5), 173.0443 (100), 163.0388 (15.1), 119.0487 (14.6), 111.0436 (3.1), 93.0329 (21.1), 85.0279 (1.6) [M‐H]^−^ 499.1252 (100), [2 M‐H]^−^ 999.2606 (2.0)	6.94	−0.090	D1	
35.	4, 5‐dicaffeoylquinic acid^a^, ^b^	C_25_H_24_O_12_ C_50_H_47_O_24_	515.1189	515.1196 (90.2), 353.0879 (100), 335.0777 (3.0), 191.0551 (50.7), 179.0339 (72.1), 173.0444 (94.8), 161.0232 (7.6), 135.0437 (70.9), 111.0439 (4.2), 93.0330 (24.7), 85.0279 (6.9) [M‐H]^−^ 515.1195 (100), [2 M‐H]^−^ 1031.25.01 (9.6)	6.97	0.137	A2	Clifford et al. (2005)
36.	4‐feruloyl‐5‐caffeoylquinic acid^a^	C_26_H_26_O_12_ C_52_H_51_O_24_	529.1356	529.1354 (85.7), 367.1034 (77.5), 193.0499 (18.2), 191.0550 (2.6), 179.0341 (0.6), 173.0444 (100), 134.0359 (15.8), 93.0329 (21.4), 85.0279 (10.3) [M‐H]^−^ 529.1359 (9.2), [2 M‐H]^−^ nd	7.09	0.009	D1	Clifford et al. (2003)
37.	4‐caffeyl‐5‐feruloylquinic acid^a^	C_26_H_26_O_12_ C_52_H_51_O_24_	529.1356	529.1357 (95.7), 367.1037 (13.9), 353.0879 (64.3), 335.0777 (1.3), 191.0551 (69.0), 191.0550 (2.6), 179.0339 (68.2), 173.0444 (100), 161.0231 (14.9), 135.0436 (70.02), 134.0359 (7.0), 111.0433 (4.2), 93.0330 (33.4), 85.0278 (7.3) [M‐H]^−^ 529.1359 (100), [2 M‐H]^−^ 1059.2826 (0.4)	7.18	1.003	D1	Clifford et al. (2003)
38.	4‐caffeyl‐5‐*p*‐coumaroylquinic acid^a^	C_25_H_24_O_11_ C_50_H_47_O_22_	499.1251	499.1248 (99.8), 353.0879 (73.0), 337.0934 (8.0), 191.0551 (65.4), 179.0338 (67.6), 173.0444 (100), 163.0387 (1.5), 161.0233 (8.2), 135.0437 (66.5), 119.0486 (0.9), 111.0435 (5.2), 93.0330 (29.9), 85.0278 (7.9)	7.62	0.471	D1	Jaiswal et al. (2010), Ren et al. (2018)
39.	3,4,5‐tricaffeoylquinic acid^a^	C_34_H_30_O_15_	677	677.1520 (100), 515.1194 (46.2), 353.0879 (47.6), 335.0772 (16.3), 191.0551 (45.1), 179.0339 (77.3), 173.0443 (90.8), 161.0232 (29.3), 135.0437 (83.5), 93.0329 (25.7), 85.0279 (6.1)	7.77	0.615	D1	Jaiswal et al. (2010)

№	Identified/tentatively annotated compound	Molecular formula	Exact mass [M‐H]^−^	Fragmentation pattern in (−) ESI‐MS/MS	*t*_R_ (min)	Δ ppm	Confidence level	References
**Caffeoylhexaric acids**								
40.	caffeoylhexaric acid 1^a^	C_15_H_16_O_11_	371.0620	371.0614 (2.7), 209.0295 (100), 191.0187 (21.6), 179.0337 (1.9), 173.0079 (0.6), 147.0287 (3.9), 135.0438 (3.6), 129.0180 (4.1), 111.0070 (2.1), 85.0278 (42.8)	1.69	−1.575	D1	Stefanova et al. (2024)
41.	caffeoylhexaric acid 2^a^	C_15_H_16_O_11_	371.0620	371.0628 (2.2), 209.0296 (100), 191.0188 (22.0), 179.0339 (1.4), 147.0283 (2.6), 135.0438 (3.4), 129.0177 (3.8), 111.0072 (2.0), 85.0278 (35.6)	2.02	−1.413	D1	
42.	caffeoylhexaric acid 3^a^	C_15_H_16_O_11_	371.0620	371.0608 (2.2), 209.0296 (100), 191.0187 (21.8), 179.0338 (1.6), 147.0285 (2.8), 135.0435 (1.0), 129.0177 (3.0), 111.0073 (2.4), 85.0279 (38.7)	2.42	−3.138	D1	
43.	caffeoylhexaric acid 4^a^	C_15_H_16_O_11_	371.0620	371.0622 (3.5), 209.0293 (100), 191.0187 (21.0), 179.0340 (1.7), 173.0078 (1.3), 147.0285 (4.1), 135.0437 (3.1), 129.0179 (3.7), 111.0071 (1.8), 85.0278 (40.4)	3.77	0.662	D1	
44.	dicaffeoylhexaric acid 1^a^	C_24_H_22_O_14_	533.0937	533.0937 (12.1), 371.0619 (85.7), 353.0511 (2.2), 209.0293 (100), 191.0187 (24.8), 179.0336 (2.8), 161.0225 (0.8), 147.0285 (3.8), 135.0437 (6.4), 129.0178 (5.9), 111.0071 (3.1), 85.0278 (50.5)	3.77	−1.504	D1	Stefanova et al. (2024), Stojakowska et al. (2016)
45.	dicaffeoylhexaric acid 2^a^	C_24_H_22_O_14_	533.0937	533.0988 (12.5), 371.0620 (85.7), 353.0505 (1.9), 209.0295 (100), 191.0189 (23.4), 179.0341 (6.5), 161.0227 (0.4), 147.0285 (3.8), 135.0437 (10.3), 129.0178 (5.9), 111.0070 (2.3), 93.0331 (0.5), 85.0278 (49.7)	4.12	−1.504	D1	Stefanova et al. (2024); Stojakowska et al. (2016)
46.	dicaffeoylhexaric acid 3^a^	C_24_H_22_O_14_	533.0937	533.0945 (41.6), 371.0643 (59.3), 353.0513 (1.9), 335.0775 (9.9), 209.0295 (100), 191.0187 (25.3), 179.0340 (18.6), 173.0444 (26.6), 161.0232 (0.7), 147.0288 (4.4), 135.0437 (78.2), 129.0179 (4.6), 111.0069 (3.6), 93.0321 (5.4), 85.0269 (44.8)	4.52	1.504	D1	Stefanova et al. (2024), Stojakowska et al. (2016)
47.	dicaffeoylhexaric acid 3^a^	C_24_H_22_O_14_	533.0937	533.0939 (13.0), 371.0616 (77.0), 353.0518 (4.9), 209.0294 (100), 191.0188 (28.5), 173.0443 (5.8), 161.0231 (1.5), 147.0285 (4.5), 135.0437 (9.1), 129.0179 (6.5), 111.0072 (3.4), 93.0332 (1.7), 85.0278 (51.3)	4.69	0.472	D1	Stefanova et al. (2024), Stojakowska et al. (2016)
48.	dicaffeoylhexaric acid 4^a^	C_24_H_22_O_14_	533.0937	533.0930 (10.5), 371.0617 (68.8), 353.0533 (2.4), 209.0294 (100), 191.0187 (23.0), 179.0337 (2.0), 161.0233 (0.7), 147.0284 (4.1), 135.0435 (3.6), 129.0180 (4.2), 111.0072 (2.4), 85.0278 (43.1)	4.90	−1.348	D1	Stefanova et al. (2024); Stojakowska et al. (2016)
49.	dicaffeoylhexaric acid 5^a^	C_24_H_22_O_14_	533.0937	533.0912 (15.3), 371.0620 (85.0), 353.0519 (2.9), 209.0295 (100), 191.0188 (24.4), 179.0338 (2.8), 173.0082 (1.2), 161.0228 (1.3), 147.0286 (4.6), 135.0436 (7.0), 129.0178 (5.3), 111.0073 (2.6), 85.0278 (48.8)	5.17	−4.555	D1	Stefanova et al. (2024), Stojakowska et al. (2016)
50.	Tricaffeoylhexaric acid‐hexoside^a^	C_39_H_38_O_22_	857.1782	857.1797 (46.8), 698.1469 (72.7), 533.1159 (16.2), 371.0616 (4.1), 341.0876 (9.9), 323.0761 (2.2), 209.0295 (100), 191.0188 (50.9), 179.0339 (27.4), 173.0079 (3.4), 161.0230 (6.5), 147.0285 (10.1), 135.0437 (40.4), 129.0178 (24.3), 111.0071 (4.4), 85.0279 (81.4)	5.22	1.813	D1	
51.	Tricaffeoylhexaric acid (leontopodic acid B)^a^, ^b^	C_33_H_28_O_17_	695.1254	695.1265 (30.2), 533.0941 (37.6), 371.0621 (20.9), 209.0294 (100), 191.0187 (16.3), 179.0338 (4.5), 161.0234 (1.4), 147.0285 (3.6), 135.0436 (6.1), 129.0178 (6.3), 111.0072 (2.0), 85.0278 (35.1)	5.89	1.565	B	Pralea et al. (2021)
52.	Tricaffeoylhexaric acid 2^a^	C_33_H_28_O_17_	695.1254	695.1255 (25.1), 533.0939 (37.2), 371.0620 (20.0), 209.0294 (100), 191.0187 (17.3), 179.0342 (2.1), 161.0232 (2.5), 147.0285 (4.5), 129.0179 (4.3), 111.0073 (2.3), 85.0278 (41.6)	6.32	0.169	D1	Pralea et al. (2021)
53.	Tricaffeoylhexaric acid 3^a^	C_33_H_28_O_17_	695.1254	695.1266 (28.2), 533.0937 (38.4), 371.0621 (20.0), 209.0295 (100), 191.0187 (17.3), 179.0341 (4.2), 173.0083 (1.2), 161.0228 (2.2), 147.0286 (4.4), 135.0437 (7.9), 129.0179 (7.4), 111.0072 (2.5), 85.0279 (46.9)	6.45	1.838	D1	Pralea et al. (2021)
54.	Hydroxybutanyl‐tricaffeoylhexaric acid (leontopodic acid A)^a^ ^,^ ^b^	C_37_H_34_O_19_	781.1622	781.1625 (49.1), 619.1310 (61.7), 457.0990 (47.7), 295.0670 (70.0), 209.0300 (4.0), 191.0187 (100), 179.0341 (11.7), 173.0079 (6.3), 161.0231 (7.0), 147.0285 (16.8), 135.0436 (15.0), 129.0179 (17.7), 111.0072 (5.3), 85.0279 (97.3)	6.53	0.420	B	Schwaiger et al. (2005)
55.	hydroxybutanyl‐tricaffeoylhexaric acid 2^a^	C_37_H_34_O_19_	781.1622	781.1632 (41.7), 619.1308 (51.5), 457.0990 (39.8), 295.0669 (63.6), 209.0298 (1.7), 191.0187 (70.3), 179.0338 (8.8), 173.0078 (8.8), 161.0233 (7.4), 147.0285 (15.4), 135.0437 (20.0), 129.0179 (14.4), 111.0073 (6.9), 85.0278 (100)	6.67	1.355	D1	Pralea et al. (2021)
56.	Hydroxybutanyl‐tricaffeoylhexaric acid 3^a^	C_37_H_34_O_19_	781.1622	781.1613 (13.6), 619.1310 (100), 457.0989 (56.6), 295.0672 (49.0), 209.0298 (3.1), 191.0188 (73.6), 179.0340 (12.3), 173.0081 (6.2), 161.0233 (6.3), 147.0285 (12.5), 135.0437 (19.6), 129.0179 (20.5), 111.0074 (6.8), 85.0278 (98.0)	6.96	−1.154	D1	Pralea et al. (2021)
57.	Hydroxyvaleryl/hydroxyisovaleryl‐tricaffeoylhexaric acid^a^	C_38_H_36_O_19_	795.1778	795.1786 (44.2), 633.1467 (42.3), 471.1147 (31.5), 309.0830 (56.6), 191.0187 (74.5), 179.0339 (9.2), 161.0230 (8.9), 147.0283 (13.9), 135.0437 (19.4), 129.0147 (15.7), 117.0542 (9.2), 111.0079 (5.9), 85.0278 (100)	6.98	0.991	D1	
58.	Hydroxybutanyl‐tricaffeoylhexaric acid 4^a^	C_37_H_34_O_19_	781.16	781.1635 (22.8), 619.1309 (76.5), 457.0986 (55.2), 295.0668 (49.2), 209.0295 (2.3), 191.0187 (71.9), 179.0339 (10.9), 173.0080 (4.8), 161.0230 (10.2), 147.0284 (14.5), 135.0436 (21.7), 129.0178 (18.4), 111.0073 (7.7), 85.0278 (100)	7.14	−1.154	D1	Pralea et al. (2021)
59.	Hydroxyvaleryl/hydroxyisovaleryl‐tricaffeoylhexaric acid^a^	C_38_H_36_O_19_	795.1778	795.1804 (11.7), 633.1465 (65.5), 471.1147 (23.9), 309.0840 (1.1), 191.0190 (16.8), 179.0338 (14.7), 147.0287 (10.5), 135.0436 (25.2), 129.0178 (24.4), 111.0069 (9.1), 85.0278 (100)	7.33	−1.197	D1	
60.	Tertracaffeoylhexaric acid 1^a^	C_42_H_34_O_20_	857.1571	857.1584 (45.5), 695.1260 (10.3), 533.0936 (5.0), 371.0021 (13.8), 209.0294 (100), 191.0187 (16.3), 179.0340 (3.5), 173.0076 (1.6), 161.0232 (5.6), 147.0285 (4.5), 135.0437 (8.1), 129.0179 (7.6), 111.0073 (2.4), 85.0278 (45.8)	7.47	1.614	D1	Pralea et al. (2021)
61.	Tertracaffeoylhexaric acid 2^a^	C_42_H_34_O_20_	857.1571	857.1588 (30.1), 695.1263 (27.9), 533.0938 (17.1), 371.0021 (14.4), 209.0295 (100), 191.0188 (16.2), 179.0341 (4.5), 173.0080 (1.5), 161.0231 (6.1), 147.0286 (4.6), 135.0437 (8.9), 129.0180 (8.8), 111.0073 (2.7), 85.0278 (41.9)	7.88	1.964	D1	Pralea et al. (2021)
62.	Butanyl/isobutanyl‐ tricaffeoylhexaric acid^a^	C_37_H_34_O_18_	765.1672	765.1683 (53.2), 603.1362 (47.4), 441.1039 (28.9), 279.0720 (83.6), 191.0187 (60.9), 179.0339 (13.4), 161.0230 (7.8), 147.0285 (9.2), 135.0436 (25.2), 129.0178 (13.0), 111.0071 (7.2), 85.0278 (100)	8.52	1.350	D1	Stefanova et al. (2024)
63.	Butanyl/isobutanyl‐ tricaffeoylhexaric acid^a^	C_37_H_34_O_18_	765.1672	765.1710 (30.0), 603.1370 (72.7), 441.1034 (48.2), 279.0720 (92.1), 191.0187 (61.2), 179.0335 (15.8), 173.0085 (3.8), 161.0234 (7.8), 147.0286 (13.3), 135.0437 (24.0), 129.0182 (14.0), 111.0075 (6.5), 85.0278 (100)	9.04	4.944	D1	Stefanova et al. (2024)
64.	2‐methylbutyl/ isovaleryl‐ tricaffeoylhexaric acid^a^	C_38_H_36_O_18_	779.1829	779.1847 (49.9), 617.1520 (42.7), 455.1198 (30.0), 293.0879 (72.8), 191.0188 (64.6), 179.0338 (13.8), 173.0080 (6.0), 161.0232 (7.0), 147.0285 (12.9), 135.0437 (24.7), 129.0179 (14.7), 111.0070 (6.2), 85.0278 (100)	9.21	−2.191	D1	Stefanova et al. (2024)
65.	Hydroxybensoyl‐ tricaffeoylhexaric acid^a^	C_40_H_32_O_19_	815.1465	815.1476 (27.4), 653.1153 (52.9), 491.0832 (48.1), 329.0513 (14.4), 209.0298 (5.5), 191.0188 (100), 179.0339 (21.2), 173.0075 (5.7), 161.0230 (12.5) 147.0286 (23.4), 137.0230 (64.7), 135.0437 (28.0), 129.0178 (19.0), 111.00072 (9.8), 93.0330 (37.7), 85.0278 (81.8)	9.24	1.396	D1	
**Carboxylic, hydroxybenzoic, and hydroxycinnamic acids, coumarins, and derivatives**								
66.	D‐mallic acid^a^, ^b^	C_4_H_6_O_5_	133.0142	133.0127 (18.4), 115.0021 (100), 89.0228 (9.7), 71.0122 (94.9)	0.75	−11.326	C	
67.	Hydroxybenzoic acid‐*O*‐hexoside 1^a^	C_13_H_16_O_8_	299.0778	299.0772 (94.3), 137.0229 (100), 109.0280 (0.3), 93.0329 (38.1)	1.30	1.335	D1	Parejo et al. (2004)
68.	Protocatechuic acid‐*O*‐hexoside^a^	C_13_H_16_O_9_	315.0727	315.0722 (100), 153.0182 (28.0), 152.0102 (58.7), 123.0071 (2.5), 109.0287 (9.0), 108.0201 (73.2)	1.69	0.269	D1	Gevrenova et al. (2022)
69.	Vanillic acid‐*O*‐hexoside^b^	C_14_H_18_O_9_	329.0875	329.0881 (1.9),167.0337 (100), 152.0102 (24.0), 123.0437 (13.5), 108.0201 (35.9)	1.77	0.804	D1	
70.	Protocatechuic acid^a^, ^b^	C_7_H_6_O_4_	153.0182	153.0180 (15.6), 109.0279 (100), 91.0174 (1.2), 81.0329 (1.5)	2.04	−8.443	B	
71.	Hydroxybenzoyl‐*O*‐hexose^a^	C_13_H_16_O_8_	299.0778	299.0776 (0.2), 239.0557 (20.5), 209.0447 (6.7), 179.0339 (47.3), 137.0229 (100), 93.0330 (19.0)	2.06	−10.167	D1	
72.	Syringic acid‐*O*‐hexoside, 1^a^	C_15_H_20_O_10_	359.0985	359.0983 (8.4), 313.0934 (26.0), 197.0446 (100), 182.0209 (17.3), 166.9976 (8.4), 153.0542 (11.5), 138.0309 (23.3), 123.0072 (44.6), 108.0201 (83.9)	2.27	−0.195	D1	Gevrenova et al. (2022)
73.	*p*‐hydroxyphenylacetic acid *O*‐hexoside 1^a^	C_14_H_18_O_8_	313.0929	313.0923 (0.4), 151.0387 (3.8), 123.0072 (19.4), 109.0282 (30.2), 108.0201 (100)	2.29	−1.427	D1	Gevrenova et al. (2022)
74.	Syringic acid‐*O*‐hexoside, 2^a^	C_15_H_20_O_10_	359.0985	359.0981 (50.4), 313.0930 (37.2), 197.0448 (89.1), 182.0211 (21.4), 166.9971 (12.9), 153.0542 (18.5), 138.0308 (29.1), 123.0073 (53.1), 108.0201 (100)	2.42	−0.640	D1	Gevrenova et al. (2022)
75.	Hydroxybenzoic acid‐*O*‐hexoside 2^a^	C_13_H_16_O_8_	299.0778	299.0773 (12.6), 137.0229 (100),93.0330 (2.3)	2.47	0.198	D1	Parejo et al. (2004)
76.	Vanillyl‐*O*‐hexose^b^	C_14_H_18_O_9_	329.0875	329.0880 (100), 269.0669 (7.2), 239.0555 (3.0), 209.0447 (34.7), 167.0337 (56.2), 152.0101 (3.7), 123.0437 (3.8), 108.0202 (3.7)	2.49	0.440	D1	Gevrenova et al. (2022)
77.	Caffeic acid‐*O*‐hexoside 1^a^	C_15_H_18_O_9_	341.0871	341.0878 (23.4), 179.0339 (19.6), 161.0231 (100), 135.0437 (6.4), 133.0280 (26.5)	2.63	−0.104	D1	Clifford et al. (2007)
78.	Aesculetin‐*O*‐hexoside^a^	C_15_H_16_O_9_	339.0724	339.0722 (23.1), 177.0181 (100), 149.0227 (0.9), 133.0380 (9.4), 105.0329 (3.9), 89.0381 (2.4)	2.69	0.250	D1	Parejo et al. (2004)
79.	Syringyl‐*O*‐hexose^a^	C_15_H_20_O_10_	359.0984	359.0985 (100), 299.0777 (4.2), 269.0667 (2.3), 239.0557 (21.8), 197.0446 (35.2), 182.0214 (3.3), 166.9970 (0.8), 153.0542 (3.4), 123.0073 (2.4)	2.77	0.390	D1	
80.	4‐hydroxybenzoic acid^a^ ^,^ ^b^	C_7_H_6_O_3_	137.0230	137.0230 (100), 109.0281 (5.0), 108.0201 (9.5), 93.0331 (2.9),65.0380 (1.4)	2.84	−10.490	B	
81.	3‐hydroxybenzoic acid^a^ ^,^ ^b^	C_7_H_6_O_3_	137.0230	137.0231 (17.8), 108.0200 (3.2), 93.0330 (100), 65.0379 (1.0)	2.95	−9.9.6	B	
82.	Gentisic acid^a^ ^,^ ^b^	C_7_H_6_O_4_	153.0182	153.0180 (47.2), 123.0072 (23.4), 109.0281 (42.9), 108.0200 (100), 81.0329 (4.7)	2.99	−8.901	B	
83.	*p*‐hydroxyphenylacetic acid *O*‐hexoside 2^a^	C_14_H_18_O_8_	313.0929	313.0930 (11.8), 151.0387 (100), 123.0074 (1.1), 109.0280 (4.1)	3.00	0.349	D1	Gevrenova et al. (2022)
84.	Hydroxybenzoic acid‐*O*‐hexoside 3^a^	C_13_H_16_O_8_	299.0778	299.0788 (3.3), 137.0229 (100), 93.0330 (48.5)	3.00	5.114	D1	Parejo et al. (2004)
85.	Caffeic acid‐*O*‐hexoside 2^a^	C_15_H_18_O_9_	341.0871	341.0878 (30.6), 179.0339 (100), 161.0231 (0.4), 135.0437 (63.9), 107.0487 (0.7)	3.09	0.279	D1	Clifford et al. (2007)
86.	Quinic acid^a^	C_7_H_12_O_6_	191.0549	191.0551 (100), 173.0444 (2.0), 155.0339 (0.4), 127.0386 (3.9), 111.0437 (1.7), 93.0330 (6.7), 85.0278 (20.4)	3.19	−5.555	D1	Ak et al. (2021)
87.	Caffeic acid‐*O*‐hexoside 3^a^	C_15_H_18_O_9_	341.0871	341.0877 (10.6), 179.0339 (100), 135.0436 (71.3), 107.0484 (0.5)	3.28	−7.081	D1	Clifford et al. (2007)
88.	Coumaric acid‐*O‐*hexoside 1^a^	C_15_H_18_O_8_	325.0930	325.0926 (10.5), 163.0388 (64.9), 145.0281 (100), 119.0487 (54.7)	3.34	−0.833	D1	Gevrenova et al. (2022)
89.	*p‐*coumaric acid^b^	C_9_H_8_O_3_	163.0389	163.0388 (6.5), 119.0487 (100), 93.0330 (1.4)	3.35	−7.590	A2	
90.	Aesculetin^a^	C_9_H_6_O_4_	177.0193	177.0181 (100), 149.0231 (3.5), 133.0280 (19.8), 105.0330 (10.2), 89.0380 (8.0)	3.47	−6.790	D1	
91.	*p*‐hydroxyphenylacetic acid^a^	C_8_H_8_O_3_	151.0401	151.0386 (100), 124.0151 (4.0), 109.0278 (14.3)	3.48	−9.451	A2	
92.	Caffeic acid^b^	C_9_H_8_O_4_	179.0339	179.0340 (18.2), 135.0437 (100), 107.0488 (1.4)	3.56	−5.485	A2	
93.	Coumaric acid‐*O‐*hexoside 2^a^	C_15_H_18_O_8_	325.0930	325.0931 (10.3), 163.0388 (11.8), 145.0280 (100), 119.0486 (3.7)	3.62	0.582	D1	Gevrenova et al. (2022)
94.	Shikimic acid^a^	C_7_H_10_O_5_	173.0455	173.0443 (100), 155.0335 (4.2), 111.0436 (100), 93.0330 (62.7)	3.84	−7.03	D1	Gevrenova et al. (2022)
95.	*m‐*coumaric acid^a^ ^,^ ^b^	C_9_H_8_O_3_	163.0389	163.0387 (7.8), 119.0486 (100), 93.0330 (1.1)	4.56	−8.326	A2	
96.	Vanillic acid^a^ ^,^ ^b^	C_8_H_8_O_4_	167.0338	167.0340 (100), 152.0101 (24.4), 123.0071 (3.9), 95.0122 (3.6)	4.79	−8.094	A2	
97.	*o‐*coumaric acid^a^ ^,^ ^b^	C_9_H_8_O_3_	163.0389	163.0388 (7.8), 119.0487 (100), 93.0329 (1.3)	5.00	−7.774	A2	
98.	Scopoletin^a^, ^b^	C_10_H_8_O_4_	191.0350	191.0340 (20.9), 176.0103 (100), 148.0152 (14.1), 120.0202 (1.4), 104.0251 (18.8)	5.06	−5.245	A2	
99.	Salicylic acid^a^, ^b^	C_7_H_6_O_3_	137.0230	137.0229 (9.6), 108.0200 (3.7), 93.0329 (100)	6.28	−10.855	A2	
**Flavonoids**								
100.	Myricetin *O*‐dihexoside^a^	C_27_H_30_O_18_	641.1359	641.1367 (100), 478.0754 (18.4), 478.0754 (18.4), 479.0829 (16.9), 317.0302 (38.5), 316.0222 (22.3), 287.0196 (10.4), 271.0251 (3.2), 243.0291 (3.2), 227.0340 (1.3), 178.9972 (1.8), 151.0027 (0.4)	3.11	1.128	D1	De Rijke et al. (2006)
101.	Patuletin *O*‐dihexoside^a^	C_28_H_32_O_18_	655.1516	655.1524 (100), 493.0992 (82.1), 535.1130 (0.5), 373.0557 (1.8), 331.0460 (55.5), 316.0215 (8.3), 287.0195 (22.7), 271.0257 (1.7), 259.0241 (8.6), 243.0298 (3.0), 215.0337 (3.4), 165.9896 (9.2), 139.0023 (5.4), 109.9996 (3.3)	3.66	1.241	D1	Ren et al. (2018)
102.	Myricetin *O*‐hexoside^a^	C_21_H_20_O_13_	479.0831	479.0833 (61.7), 317.0301 (100), 287.0205 (0.6), 271.0255 (2.3), 243.0294 (1.0), 227.0338 (0.9), 199.0390 (1.1), 178.9978 (1.6), 151.0022 (0.9)	4.55	0.284	D1	De Rijke et al. (2006)
103.	Rutin^b^	C_27_H_30_O_16_	609.1461	609.1466 (100), 301.0351 (30.8), 300.0275 (56.0), 271.0247 (26.7), 255.0297 (12.0), 211.03908 (0.5), 178.9970 (2.9), 151.0024 (5.3), 121.0280 (0.7), 107.0122 (1.4)	5.09	0.824	B	
104.	Myricetin 3‐*O*‐acetylhexoside^a^	C_23_H_22_O_14_	521.0937	521.0937 (74.3), 317.0301 (100), 316.0224 (27.5), 287.0197 (1.6), 271.0246 (2.9), 243.0293 (1.7), 227.0342 (1.0), 178.9979 (1.8), 151.0024 (0.9), 107.0125 (0.2)	5.21	0.022	D1	Ren et al. (2018)
105.	Patuletin 3‐*O*‐rutinoside^a^	C_28_H_32_O_17_	639.1567	639.1581 (100), 331.0453 (29.1), 330.0381 (58.1), 316.0223 (11.1), 315.0152 (17.1), 287.0201 (15.6), 271.0245 (4.0), 259.0250 (3.9), 243.0292 (3.6), 215.0344 (3.7), 165.9897 (4.1), 139.0386 (0.8), 149.0229 (6.3), 109.9998 (0.9)	5.23	2.202	D1	Ren et al. (2018)
106.	Quercetin 4′‐*O*‐hexoside^a^	C_21_H_20_O_12_	463.0882	463.0885 (48.1), 301.0352 (100), 300.0276 (24.0), 271.0247 (12.2), 255.0298 (6.6), 227.0348 (2.2), 178.9979 (1.1), 151.0024 (2.5), 121.0280 (0.7), 107.0123 (0.8)	5.30	0.714	D1	
107.	6‐hydroxyluteolin‐malonylhexoside^a^	C_24_H_22_O_15_	549.0886	549.0877 (5.6), 505.0993 (100), 463.0893 (1.9), 343.0435 (0.3), 300.0276 (28.8), 301.0352 (79.8), 271.0245 (1.1), 255.0294 (2.6), 243.0294 (1.0), 227.0342 (3.2), 175.0384 (0.9), 215.0343 (1.2), 199.0392 (2.1), 165.9892 (0.9), 136.9866 (8.6), 133.0278 (2.9)	5.36	−1.608	D1	
108.	Luteolin 7‐*O*‐glucoside^a^, ^b^	C_21_H_20_O_11_	447.0933	447.0935 (100), 327.0509 (1.6), 285.0402 (88.9), 284.0326 (34.9), 227.0341 (2.4), 151.0023 (4.9), 133.0281 (4.2), 107.0122 (3.3)	5.39	0.213	B	
109.	Patuletin 3‐*O*‐hexoside^a^	C_22_H_22_O_13_	493.0988	493.0988 (30.9), 331.0461 (100), 316.0225 (10.3), 287.0197 (2.5), 271.0251 (1.6), 243.0299 (1.1), 227.0339 (0.7), 259.252 (0.4), 215.0341 (0.4), 165.9895 (10.9), 139.0024 (7.8), 136.9870 (0.9), 109.9994 (5.3), 178.9976 (1.3)	5.53	0.114	D1	Ren et al. (2018)
110.	Nepetin 7 *O*‐glucoside^a^, ^b^	C_21_H_22_O_12_	477.1038	477.1038 (100), 462.0807 (8.7), 315.0509 (69.3), 314.0434 (36.4), 300.0272 (14.4), 299.0197 (19.8), 297.0411 (0.6), 271.0248 (35.7), 243.0295 (3.2), 199.0393 (4.8), 136.9866 (5.4), 109.9995 (0.5)	5.55	−0.061	B	
111.	Kaempferol 3‐*O*‐rutinoside^a^ ^,^ ^b^	C_27_H_30_O_15_	593.1512	593.1516 (100), 285.0402 (68.8), 284.0326 (39.0), 255.0296 (30.4), 227.0343 (20.7), 211.0388 (1.8), 151.0022 (1.6), 135.0073 (0.9), 107.0123 (1.5)	5.64	0.703	B	
112.	isorhamnetin 3‐*O*‐rutinoside^a^ ^,^ ^b^	C_28_H_32_O_16_	623.1618	623.1619 (100), 315.0510 (80.8), 314.0434 (32.1), 300.0273 (12.9), 271.0247 (28.3), 255.0296 (12.0), 243.0294 (16.5), 227.0342 (4.5), 199.0391 (4.7), 161.0231 (3.8), 151.0022 (3.8), 107.0122 (0.7)	5.78	0.276	B	
113.	Luteolin 7‐*O*‐dihexoside^a^	C_27_H_30_O_16_	609.1461	609.1467 (100), 447.0899 (0.7), 429.0830 (10.7), 285.0403 (76.9), 256.0374 (8.3), 241.0500 (0.7), 151.0023 (6.4), 133.0280 (8.3), 107.0123 (1.8)	5.95	1.021	D1	De Rijke et al. (2006)
114.	Quercetin *O*‐acetylhexoside^a^	C_23_H_22_O_13_	505.0988	505.0993 (17.7), 343.0445 (0.3), 301.0353 (100), 271.0246 (1.0), 245.0453 (21.6), 227.0341 (1.3), 178.9971 (0.3), 151.0022 (0.7)	6.01	1.002	D1	
115.	Apigenin 7‐*O*‐glucoside^b^	C_21_H_20_O_10_	431.0984	431.0982 (100), 341.0673 (0.2), 311.0552 (1.2), 269.0448 (28.6), 268.0375 (62.1), 240.0423 (4.2), 211.0393 (1.7), 151.0023 (3.5), 117.0330 (1.3), 107.01241 (1.8)	6.07	−0.371	B	
116.	Patuletin *O*‐acetylhexoside^a^	C_24_H_24_O_14_	535.1093	535.1091 (25.3), 373.0568 (0.6), 331.0460 (100), 330.0376 (0.5), 316.0225 (10.9), 287.0192 (1.6), 271.0244 (0.9), 259.0192 (1.6), 243.0295 (0.5), 165.9895 (8.8), 139.0021 (8.0), 136.9866 (0.5), 178.9977 (1.8), 151.0021 (2.9)	6.14	−0.487	D1	
117.	Luteolin *O*‐acetylhexoside^a^	C_23_H_22_O_12_	489.1038	489.1039 (100), 447.0938 (0.6), 429.0836 (0.4), 327.0500 (1.2), 297.0408 (0.2), 285.0401 (75.3), 284.0326 (40.9), 256.0374 (4.1), 227.0346 (2.2), 211.0396 (1.2), 175.0391 (1.4), 151.0023 (5.4), 133.0280 (4.5), 107.0123 (3.6)	6.18	0.002	D1	
118.	Luteolin 4′ *O*‐hexoside^a^	C_21_H_20_O_11_	447.0933	447.0935 (53.4), 285.0403 (100), 256.0378 (2.5), 241.0501 (0.8), 217.0496 (1.5), 151.0024 (6.4), 133.0280 (11.8), 107.0123 (2.5)	6.42	0.571	D1	
119.	luteolin 7‐*O*‐pentosyl‐hexoside^a^	C_26_H_28_O_15_	579.1355	579.1358 (100), 447.0917 (2.5), 429.0826 (13.1), 285.0404 (86.4), 284.0324 (18.3), 269.0473 (0.5), 256.0374 (11.2), 217.0501 (1.6), 151.0024 (5.3), 133.0279 (7.4), 107.0121 (2.1)	6.66	0.443	D1	
120.	Patuletin^a^	C_16_H_12_O_8_	331.0459	331.0459 (100), 316.0223 (40.9), 287.0190 (1.1), 271.0252 (1.5), 259.0236 (0.4), 243.0291 (0.9), 181.0132 (14.1), 165.9895 (17.3), 139.0022 (9.3), 136.9866 (1.4), 121.0280 (5.5), 109.9993 (12.3)	6.82	−0.032	D1	Ren et al. (2018)
121.	Apigenin 7‐*O*‐acetylhexoside^a^	C_23_H_22_O_11_	473.1089	473.1093 (100), 413.0864 (0.3), 311.0559 (1.5), 283.0612 (0.3), 269.0447 (24.9), 268.0376 (63.3), 240.0423 (4.8), 211.0397 (2.4), 151.0023 (3.8), 117.0327 (2.1), 107.0122 (2.8)	6.89	0.857	D1	
122.	Luteolin^b^	C_15_H_10_O_6_	285.0405	285.0403 (100), 267.0313 (0.2), 243.0295 (0.3), 217.0506 (1.3), 199.0396 (2.4), 175.0388 (3.0), 151.0023 (4.8), 143.0492 (0.2), 133.0280 (24.1), 107.0122 (3.9)	7.59	−0.741	A2	
123.	Quercetin^a^ ^,^ ^b^	C_15_H_10_O_7_	301.0354	301.0353 (100), 273.0407 (2.9), 257.0485 (1.8), 245.0451 (1.3), 229.0496 (1.2), 211.0388 (0.9), 178.9975 (21.3), 151.0023 (45.1), 121.0280 (13.2), 107.0123 (13.9)	7.61	−0.219	A2	
124.	Nepetin^a^ ^,^ ^b^	C_16_H_12_O_7_	315.0510	315.0509 (100), 300.0273 (38.6), 287.0560 (11.5), 227.0338 (0.8), 165.9895 (23.9), 139.0023 (7.2), 136.9863 (2.6), 133.0281 (1.1), 109.9993 (14.3)	8.01	−0.304	A2	
125.	Apigenin 7‐*O*‐caffeoyl‐hexoside^a^	C_30_H_26_O_13_	593.1301	593.1307 (100), 431.0978 (3.9), 413.0873 (1.3), 269.0455 (58.4), 179.0338 (9.7), 161.0230 (62.3), 135.0437 (12.1), 117.0328 (5.9)	8.40	1.140	D1	
126.	Kaempferol^b^	C_15_H_10_O_6_	285.0405	285.0403 (100), 267.1601 (0.3), 257.0459 (1.6), 241.0503 (1.2), 227.0349 (0.6), 211.0394 (1.2), 161.0238 (0.4), 135.0072 (1.3), 151.0022 (3.1), 107.0122 (1.9)	8.85	−0.425	A2	
127.	Apigenin^a^ ^,^ ^b^	C_15_H_10_O_5_	269.0455	269.0454 (100), 225.0544 (1.5), 183.0444 (0.5), 151.0022 (5.1), 117.0330 (17.6), 107.0121 (4.9)	8.63	−0.508	A2	
128.	kaempferide/isokaempferide^a^	C_16_H_12_O_6_	299.0561	299.0559 (100), 284.0325 (83.5), 256.0374 (19.7), 227.0343 (2.9), 211.0394 (0.8), 188.0467 (0.3), 151.0024 (3.2), 132.0206 (0.6), 107.0121 (1.9)	8.93	−0.473	D1	Ren et al. (2018)
129.	Isorhamnetin^a^, ^b^	C_16_H_12_O_7_	315.0510	315.0512 (100), 300.0274 (42.7), 271.0251 (2.8), 255.0298 (2.0), 227.0341 (1.8), 164.0100 (2.6), 151.0022 (7.8), 107.0122 (8.7)	9.12	0.584	A2	
130.	Genkwanin^a^, ^b^	C_16_H_12_O_5_	283.0612	283.0610 (100), 268.0376 (51.3), 239.0348 (3.7), 151.0022 (2.5), 117.0330 (3.8), 107.01222 (1.9)	11.62	−0.554	A2	

*Note:* [M‐H]^−^ ‐deprotonated molecular ion; Exact mass: calculated mass of an ion whose elemental formula, isotopic composition and charge state are known, that is, it is the theoretical mass (Brenton and Godfrey 2010); *t*_R_: retention time; Δ ppm: delta parts per million‐a measurement of the mass accuracy, or the difference between an experimentally measured mass and its theoretically calculated mass; Confidence level: A2: confirmed structure including confirmed stereochemistry; B: confirmed structure except for one or more stereochemical aspects; C: tentative identification matched with a standard compound, match of at least *t*_R_, MS and MS/MS with an actual authentic standard analyzed in parallel, preferably supported by other online data; D: Tentative identification based on libraries, model compounds etc.; D1: relatively reliable evidence; D2: relatively poor evidence; E: tentative candidate or tentative identification of metabolite class (Cicek et al. 2012).

^a^ Reported for the first time.

^b^ Compare to reference standard.

#### 3.1.1 Acylquinic Acids

Herein, 16 *mono*AQA and 10 *di*AQA acids together with 1 *tri*acylquinic acid were dereplicated or annotated in the *A. dioica* extract (Table 1, Figure S1).

The AQAs annotation was based on the characteristic fragment ions and their relative abundances ascribed to each subclass AQAs. The Orbitrap high resolution mass spectrometry strategy was developed on the base of Clifford's and Jaiswal's hierarchical keys for identification of chlorogenic acids (Clifford et al. 2003, Clifford et al. 2005; Jaiswal et al. 2010), as well as data acquired by quadrupole‐Orbitrap mass spectrometry reported elsewhere (Gevrenova et al. 2020; Ren et al. 2018). In Full MS scan mode, AQAs yielded typically deprotonated molecules [M‐H]^−^ together with the dimeric [2 M‐H]^−^ at the corresponding retention time of each compound. For instance, at 3.19 min, ions at *m*/*z* 353.087 [M‐H]^−^ (calc. for C_16_H_17_O_9_) (100%) and 707.183 [2 M‐H]^−^ (calc. for C_32_H_35_O_18_) (74%) were registered in the extracted ion chromatogram of caffeoylquinic acids at *m*/*z* 353.087 (exact mass) with mass accuracy 5 ppm (Figure S2). In the same way, [2 M‐H]^−^ at *m*/*z* 675.195 (calc. for C_32_H_35_O_16_), 735.216 (calc. for C_34_H_39_O_16_), and 1031.249 (calc. for C_50_H_47_O_24_) were acquired for *p*‐coumaroylquinic, feruloylquinic, and dicaffeoylquinic acid, respectively (Table 1). The fragmentation pattern of quinic acid (QA) was delineated by the subsequent transitions: *m/z* 191.055 [QA‐H]^−^ → 173.044 [QA‐H‐H_2_O]^−^ → 111.043 [QA‐H‐2H_2_O‐CO_2_]^−^ → 93.033 [QA‐H‐3H_2_O‐CO_2_]^−^ and *m/z* 85.028 [QA‐H‐2H_2_O‐CO‐C_2_H_2_O]^−^ (Clifford et al. 2003). Key points in the AQAs annotation are the fragment ions at *m/z* 179.034 (caffeoylquinic acids, CQAs), 193.050 (feruloylquinic acids, FQAs), 163.039 (p‐coumaroylquinic acids, *p*‐CoQAs), and 197.045 (hydroxydihydrocaffeoylquinic acids, HCQAs).

The substitution at C‐5 of quinic acid skeleton was indicated by the base peaks at *m/z* 191.055 [QA‐H]^−^. Accordingly, **2, 7/11, 8, 12/17** and **15/19** were assigned as 5‐hydroxydihydrocaffeoyl, 5‐caffeoyl‐, 5‐dihydrocaffeoyl, 5‐*p*‐coumaroyl and 5‐feruloylquinic acid and their isomers, respectively (Figure S3). Compounds **4**, **6** and **10** were ascribed to 3‐substituted quinic acid conjugates, evidenced by the base peaks at *m/z* 191.055 (**4**), 163.039 (**6**) and 193.050 (**10**) alongside *m/z* 135.044 [caffeic acid‐H‐CO_2_]^−^, 119.049 [*p*‐coumaric acid‐H‐CO_2_]^−^ and 134.036 [ferulic acid‐H‐CO_2_‐CH_3_]^−^, respectively. (Figure S4). The 4‐substituted *mono*AQAs **3**, **5**, **9** and **18** were deduced from the base peak at *m/z* 173.044 corresponding to the “dehydrated” quinic acid ion at *m/z* 173.044. Four commonly found in Asteraceae family *di*AQA subclasses were dereplicated/annotated as follows: dicaffeoylquinic acid (*di*CQA) isomers at *m*/*z* 515.120 [M‐H]^−^, feruloyl‐caffeolylquinic acids (F‐CQA) at *m*/*z* 529.135, *p*‐coumaroyl‐caffeoylquinic acids (*p*‐Co‐CQA) at *m*/*z* 499.125 and hydroxydihydrocaffeoyl‐caffeolylquinic acids (HC‐CQA) at *m/z* 533.130 alongside their dimeric adducts (Table 1, Figure S5). Fragmentation pattern of six isobaric peaks (**20**–**24** and **35**) yielded transitions at *m*/*z* 515.120 → 353.088 → 191.055, suggesting consequent losses of two caffeoyl residues (162.03 Da) (Table 1). The base peak at *m*/*z* 191.055 (**22** and **23**) pointed out on the substitution at C‐1 (3) and C‐5 of the quinic acid skeleton, as was seen in neochlorogenic and chlorogenic acid (Figures S3, S4 and S6). 3, 5‐*di*CQA was evidenced by the abundant ions at *m*/*z* 179.034 [caffeic acid‐H]^−^ and 135.044 [caffeic acid‐H‐CO_2_]^−^ alongside negligible ion at *m*/*z* 335.080 [M‐H‐caffeoyl‐H_2_O]^−^. Regarding the isobaric **28**, **29** and **30** ([M‐H]^−^ at *m*/*z* 499.125), the relative abundances of the diagnostic ions at *m*/*z* 337.093 [M‐H‐caffeoyl]^−^ and 353.085 [M‐H‐*p*‐coumaroyl]^−^ are relevant for the assignment of 3, 5 *di*AQAs. Accordingly, the ion at *m*/*z* 337.093 [M‐H‐caffeoyl]^−^ (76.4%) accompanied by *m*/*z* 163.039 [*p*‐coumaric acid‐H]^−^ (base peak) suggested 3‐*p*‐Co‐5‐CQA (**28**), while 3‐C‐5‐*p*‐CoQA (**29**/**31**) was discernable by the indicative ions at *m*/*z* 353.085 [M‐H‐*p*‐coumaroyl]^−^ (67.1%) and *m*/*z* 191.055 (100%). In the same way, **32** and **33** were ascribed to 3‐F‐5‐CQA and 3‐C‐5‐FQA by the base peaks at 193.050 and 191.055, respectively, corroborated by the fragment ions at *m*/*z* 367.103 (33.8%) (**32**) and 353.088 (39.6%) (**33**).

**FIGURE 2:**
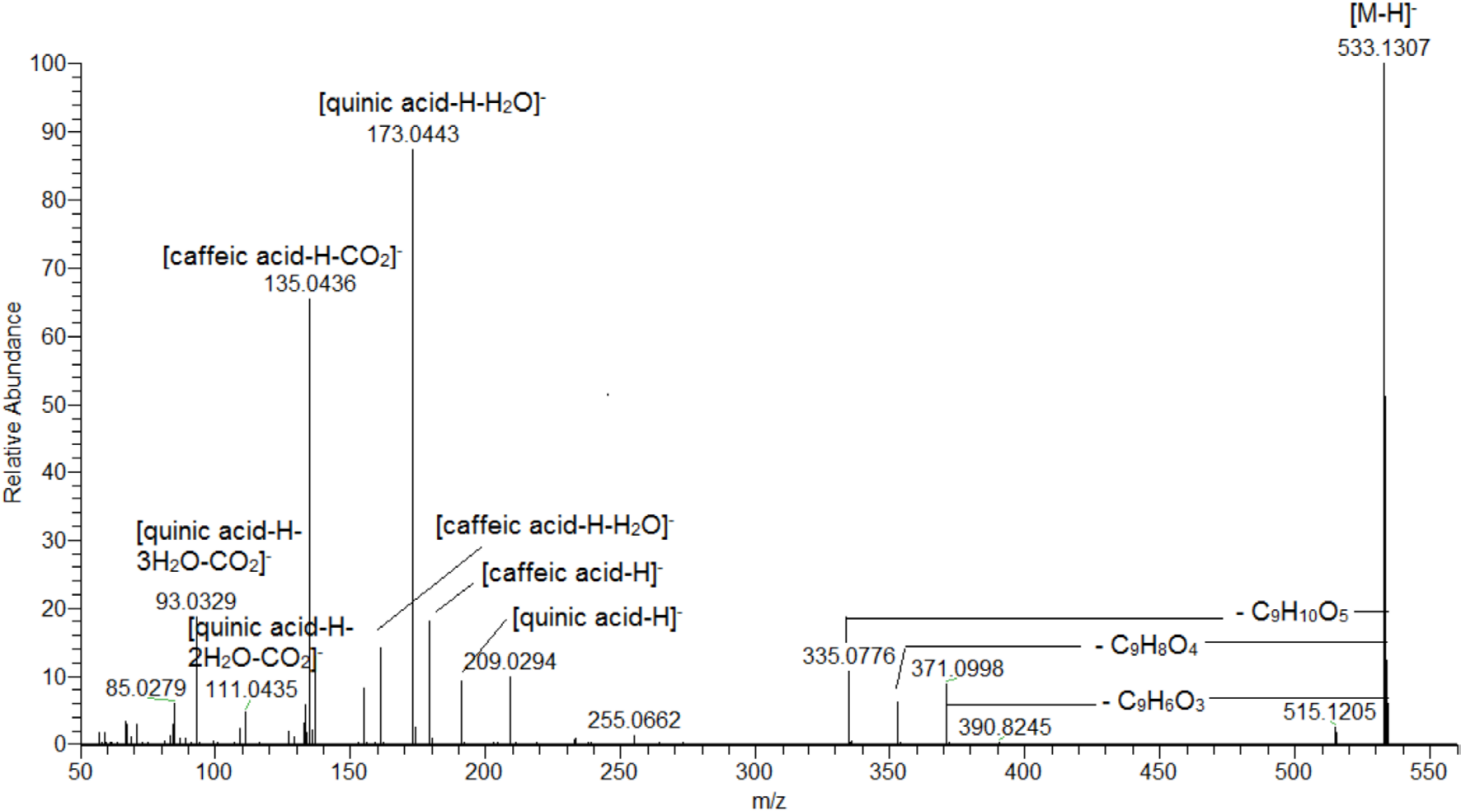
MS/MS spectrum of 3‐caffeoyl‐4‐ hydroxy‐dihydrocaffeoylquinic acid (**14**).

Vicinal *di*acylquinic acids (**14**, **21, 24, 26, 27**, **30, 34, 35, 36**, **37**, and **38**) along with 3,4,5‐*tri*caffeoylquinic acid (**39**) were evidenced by the diagnostic “dehydrated” quinic acid ion at *m/z* 173.045 (Table 1, Figure 2, Figures S7–S11) (Clifford et al. 2003). Exemplified by **34** ([M‐H]^−^ at *m/z* 499.125), the relative abundances of the prominent ions at *m/z* 353.087 [M‐H‐*p*‐coumaroyl]^−^ (2.4%) and 337.093 [M‐H‐caffeoyl]^−^ (68.1%) indicated a loss of the caffeoyl moiety before the *p*‐coumaroyl one. Owing to the fact that **34** yielded a base peak at *m/z* 173.044 (100%), consistent with a C‐4 substituted quinic acid backbone, accompanied by *m/z* 163.039 and 119.049, it was assigned as 4‐*p*‐Co‐5‐CQA.

Neochlorogenic (**4**), chlorogenic (**7**), 3,4‐ (**21**), and 1, 5‐dicaffeoylquinic (**23**) were unambiguously identified by comparison with reference standards (Figures S3, S4, S6 and S7). The extracted ion chromatograms of AQAs revealed that the *A. dioica* profile was dominated by chlorogenic acid (**7**) (18.36%), 3,5‐dicaffeoylquinic acid (**22**) (17.79%), neochlorogenic acid (**4**) (12.17%), together with 4,5‐ dicaffeoylquinic acid (**24**) (12.17%), and 3,4‐dicaffeoylquinic acid (**21**) (7.77%) (Figure S1). The comparison of the relative abundance and percentage ratio of all annotated compounds reveled that AQAs were the main classes secondary metabolites found in *A. dioica* aerial parts (55.62%).

#### 3.1.2 Caffeoylhexaric Acids (CHAs)

**FIGURE 3:**
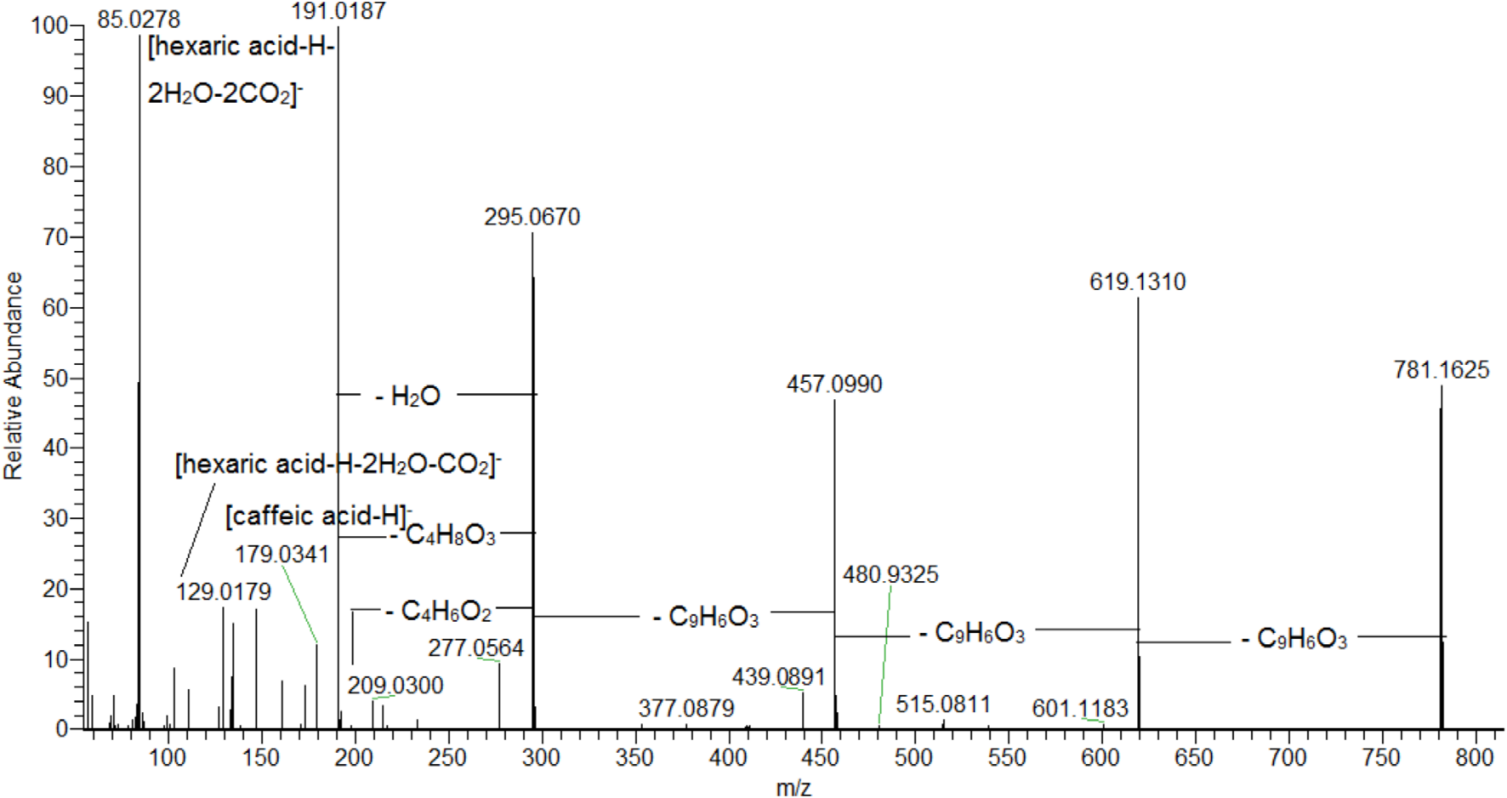
MS/MS spectrum of leontopodic acid A (**54**).

Generally, 4 *mono*‐, 6 *di*‐, 3 *tri*‐ and 2 *tetra*caffeoylhexaric acids were annotated together with *tri*CHA esters with aliphatic acids (**54**–**59**, **62**–**65**) (Table 1, Figure S12). In the fragmentation pattern of acylhexaric acids, the deprotonated molecule of hexaric acid (HA) at *m*/*z* 209.026 (C_6_H_9_O_8_) was commonly found, corroborated by the fragment ions at *m*/*z* 191.019 [HA‐H‐H_2_O]^−^, 173.008 [HA‐H‐2H_2_O]^−^, 147.029 [HA‐H‐H_2_O‐CO_2_]^−^, 129.018 [HA‐H‐2H_2_O‐CO_2_]^−^, 111.007 [HA‐H‐3H_2_O‐CO_2_]^−^, 85.028 [HA‐H‐2H_2_O‐2CO_2_]^−^(**40**) (Table 1, Figure 3). A variety of CHAs at *m*/*z* 371.062 [M‐H]^−^ (**40**–**43**) (Figure S13), dicaffeoylhexaric acids *m*/*z* 533.094 (**44**–**49**) (Figure S14), tricaffeoylhexaric acids at *m*/*z* 695.125 (**51–53**) (Figure S15) and tetracaffeoylhexaric acids at *m*/*z* 857.157 (**60, 61**) (Figure S16), were annotated in the assayed extract. *Mono*CHAs acid isomers were dedicated from the loss of caffeoyl residue (162.03 Da) at *m*/*z* 209.030 [M‐H‐caffeoyl]^−^. Caffeoyl residue (CA) typically gave the fragment ions at *m*/*z* 179.034 [CA‐H]^−^, 161.023 [(CA‐H)‐H_2_O]^−^ and 135.044 [(CA‐H)‐CO_2_]^−^. *Di*CHAs afforded subsequent losses of 2 CA residues at *m*/*z* 371.062 and 209.030, whereas in *tri*CHAs the losses of 3 CA residues were registered at *m*/*z* 533.094, 371.062 and 209.030, respectively (Pralea et al. 2021). Thus, on the base of comparison with reference standard, compound **51** was identified as leontopodic acid B. The *tetra*CHAs were assigned on the base of the following transitions: 857.158 → 695.126 → 533.094 → 371.062 → 209.030 (Pralea et al. 2021). Compounds **54–56** and **58** shared the same [M‐H]^*−*^ at *m*/*z* 781.162 (calc. for C_37_H_33_O_19_). They yielded the prominent fragment ions at *m*/*z* 295.067 [M‐H‐3CA]^−^, 209.030 [M‐H‐3CA‐C_4_H_6_O_2_]^−^ and 191.019 [M‐H‐3CA‐C_4_H_8_O_3_]^−^ resulting from the losses of 3 CA residues and a subsequent loss of a hydroxybutanyl group 86.037 Da (calc. for C_4_H_6_O_2_) and hydroxybutyric acid (C_4_H_8_O_3_), respectively (**54**) (Figure 3). Consequently, **54–56** and **58** were assigned as isomeric hydroxybutanyl‐tricaffeoylhexaric acid (Table 1) (Schwaiger et al. 2005). This assignment is consistent with the structure of leontopodic acid A, additionally confirmed for **54** by comparison with a reference standard (Figure 3).

The fragmentation pathways of **57** and **59** were delineated by the following transitions: *m*/*z* 795.179 → 633.147 → 471.115 → 309.083 → 191.019, suggesting a loss of three caffeoyl moieties (3 × 162 Da) and 118.064 Da (calc. for C_5_H_10_O_3_) consistent with hydroxyvaleric/hydroxyisovaleric acid (Table 1, Figure S17). Accordingly, **57** and **59** were assigned as isomeric hydroxyvaleryl/hydroxyisovaleryl‐tracaffeoylhexaric acid. Regarding **62** and **63** ([M‐H]^−^ at *m*/*z* 765.168), either butanyl or isobutanyl moiety was deduced from the prominent ion at *m*/*z* 279.072 [M‐H‐3CA]^−^ and 191.034 [M‐H‐3CA‐C_4_H_8_O_2_]^−^ (Figure S18) (Stefanova et al. 2024). In the same manner, **64** with [M‐H]^−^ at *m*/*z* 779.185 was annotated as methylbutanyl/isovaleryl‐tricaffeoyl‐hexaric acid. A methylbutanyl or isovaleryl moiety was evidenced by the loss of 102.069 Da (C_5_H_10_O_3_) and three CA residues at *m*/*z* 191.019 (**64**). A hydroxybenzoyl moiety was deduced in **65** ([M‐H]^−^ at *m*/*z* 815.148), additionally supported by the prominent ions at *m*/*z* 137.023 [hydroxybenzoic acid (HBA)‐H]^−^ and 93.033 [HBA‐H‐CO_2_]^−^. The structure of compounds **51** (leontopodic acid B) and **54** (leontopodic acid A) was unambiguously confirmed by comparison with reference standards. Both compounds are caffeoyl‐D‐glucaric acid derivatives and were previously found in other Gnaphalieae species, like alpine edelweiss (*Leontopodium alpinum*) and several *Gnaphalium* species from the Alps (Cicek et al. 2012). Additionally, 2,5‐dicaffeoylglucaric acid, 3,4‐dicaffeoylglucaric acid, and 2,4‐ or 3,5‐dicaffeoylglucaric acid were previously isolated from *Eupatorium perfoliatum* L.; Asteraceae (Maas et al. 2008). Four isomer dicaffeoylhexaric acids were isolated from *Berberis microphylla* G. Forst (Berberidaceae), two of them have been assigned as 3‐ and 4‐transcaffeoyl‐glucaric acids, but the determination of the linkage position for each isomer was not possible. A third isomer was also isolated, but it easily degraded and was suggested to be the 2‐ or 5‐trans‐caffeoyl‐glucaric acid (Ruiz et al. 2014). From the aerial parts of *Galinsonga parviflora* (Asteraceae), 2,3,4,5‐tetracaffeoylglucaric acid and 2,4,5‐tricaffeoylglucaric acid were previously isolated (Dudek et al. 2016).

Herein, among CHAs, leontopodic acid A (**54**) (25.96%), followed by leontopodic acid B (**51**) (12.59%), together with its isomer tricaffeoylhexaric acid (**52**) (11.97%) appeared to be dominant for *A. dioica* extract.

#### 3.1.3 Hydroxybenzoic and Hydroxycinnamic Acids and Derivatives

The compounds **67**–**69**, **72, 74, 75** and **84** were closely associated to the fragmentation patterns of hydroxybenzoic acid—hexosides, affording prominent fragment ions at *m/z* 137.023 [(M‐H)‐Hex]^−^(**67**, **75**, **84**), 153.018 (**68**), 167.034 (**69**) and 197.045 (**72** and **74**), which are consistent with hydroxybenzoic, protocatechuic, vanillic and syringic acid, respectively (Gevrenova et al. 2022) (Table 1, Figure S19). Likewise, **77**, **85, 87, 88** and **93** could be assigned as hydroxycinnamic acid‐hexosides, including caffeic acid‐hexosides (**77**, **85** and **87**), and coumaric acid‐hexosides (**88** and **93**), evidenced by the indicative fragment ions at *m/z* 179.034 [CA‐H]^−^ and 163.039 [coumaric acid‐H]^−^, respectively (Clifford et al. 2007; Gevrenova et al. 2022). (−) ESI‐MS/MS spectra of the sugar esters hydroxybenzoyl‐hexose (**70**), vanillyl‐hexose (**75**) and syringoyl‐hexose (**78**) were obtained. Ester bonded hydroxybenzoic acids were evidenced by the hexose cross ring cleavages ^0,4^Hex (−60 Da), ^0,3^Hex (−90 Da) and ^0,2^Hex (−120 Da) (Clifford et al. 2007). Based on the comparison with reference standards, five hydroxybenzoic acids (**70**, **80**–**82** and **99**) and 5 hydroxycinnamic acids (**89**, **90**, **95–97**) together with mallic (**66**), quinic (**86**), *p*‐hydroxyphenylacetic (**92**) and shikimic (**94**) acid were identified in the extract (Table 1). MS/MS spectra of the identified compounds are depicted in Figures S20–S32.

Among the hydroxybenzoic and hydroxycinnamic acids and derivatives, being present at 21.70%, the *A. dioica* extract was dominated by protocatechuic acid *O*‐hexoside (**68**) (16.47%), syringic acid *O*‐hexoside (**72**) (12.36%), and quinic acid (**86**) (11.94%).

#### 3.1.4 Flavonoids

Flavonoids annotated in the studied extract belong to the class of flavones and flavonols. They mainly comprise apigenin, luteolin, nepetin, kaempferol, quercetin, myricetin, isorhamnetin, and patuletin as aglycone moiety and their mono‐, di‐, and acetylglycosides (Table 1, Figure S33). The strategy for the flavonoid dereplication was based on the fragmentation pathways and diagnostic fragments corresponding to different classes of flavonoids (Gevrenova et al. 2020). A series of flavonoids were closely related to the same fragmentation pattern generating characteristic fragments at *m/z* 317.030 (**102**), 301.0352 (**106**), 285.041 (**108**), 331.046 (**109**), 315.051 (**110**) [(M‐H)‐Hex]^−^, indicating hexoside of myricetin, quercetin, luteolin, patuletin, and nepetin, respectively (Gevrenova et al. 2022; Ren et al. 2018) (Table 1). Compounds **100**, **101**, **113** gave neutral loss of 324.106 Da [M‐H‐2Hex]^−^, while compounds **103**, **105**, **111**, **112** afforded neutral loss of 308.112 Da [M‐H‐rut]^−^ consistent with dihexosides and rutinosides, respectively.

Based on the comparison with retention times and MS/MS fragmentation pathways of reference standards, **103, 108, 110, 111, 112, 122, 123, 124, 126, 127, 129, 130** were unambiguously identified as rutin, luteolin 7‐*O*‐glucoside, nepetin 7‐*O*‐glucoside, kaempferol 3‐*O*‐rutinoside, isorhamnetin 3‐*O*‐rutinoside, luteolin, quercetin, nepetin, kaempferol, apigenin, isorhamnetin, and genkwanin, respectively. The MS/MS spectra of the aforementioned flavonoids are depicted in the Figures S34–S46. Compound **104** gave a precursor ion at [M‐H]^−^ at *m/z* 521.094 (C_23_H_22_O_14_) together with a fragment ion at *m/z* 317.030, [M‐H‐204.064 Da]^−^, corresponding to the concomitant loss of hexose and acetyl residue. Thus, **104** was annotated as myricetin 3‐*O*‐acetylhexoside. Regarding 128 ([M‐H]^−^ at *m/z* 299.056, C_16_H_11_O_6_), unsubstituted A‐ring was assumed from the RDA ions at *m/z* 151.002 (^1,3^A^−^) and 107.012 (^0,4^A^−^), while the fragment at *m/z* 132.020 (^1,3^B^−^) pointed out on a methoxylation in B‐ring, as was presented in kaempferide/isokaemferide (De Rijke et al. 2006). Compound **125** was related to caffeoyl ester of apigenin 7‐*O*‐hexoside (Table 1). The losses of 162.030 Da (C_9_H_6_O_3_) and 324.0852 Da (C_15_H_16_O_8_) in the fragmentation pathway, together with the characteristic ions at *m/z* 179.034 [caffeic acid‐H]^−^, 161.023 [caffeic acid‐H‐H_2_O]^−^, and 135.044 [caffeic acid‐H‐CO_2_]^−^ corroborated caffeoyl residue. Apigenin‐7‐*O*‐(6″‐*O*‐E‐caffeoyl)‐*β*‐D–glucopyranoside was previously isolated from *Chamomilla recutita* [L.] Rauschert (Švehlíková et al. 2004) and *Leucas aspera* L. (Manivannan 2016).

Within this group it should be noted the dominance of myricetin *O*‐hexoside (**102**) (13.99%), luteolin 7‐*O*‐glucoside (**108**) (11.05%) and luteolin (**122**) (10.88%). The relative quantity of flavonoids reached to 6.85%.

### 3.2 Total Bioactive Compounds, Antioxidant and Enzyme Inhibitory Properties of *A. dioica* Extract

The assessment of total phenolic and flavonoid contents was performed using spectrophotometric methods (Table 2). The obtained results revealed the higher total phenolic content (77.7 ± 0.1 mg GAE/g) than previously reported by Babotă et al. (2018), where *A. dioica* flowers contain 19.62 mg GAE/g (MeOH extract), 14.01 mg GAE/g (EtOH extract), and 36.27 mg GAE/g (70% EtOH extract) polyphenols (Babotă et al. 2018). Regarding the total flavonoid content, our results are in line with those previously found in the ethanol extract (11.95 mg QE/g), but lower than in methanol (21.38 mg QE/g) and 70% ethanol (21.88 mg QE/g) extracts (Babotă et al. 2018).

**TABLE 2:** Total phenolic and flavonoid contents, antioxidant and enzyme inhibitory properties of *A. dioica* extract.

**Total bioactive compounds**	
Total phenolic content (mg GAE/g)	77.7 ± 0.1
Total flavonoid content (mg RE/g)	11.8 ± 0.2
**Antioxidant properties**	
DPPH scavenging ability (mg TE/g)	53.8 ± 0.1
ABTS scavenging ability (mg TE/g)	141.6 ± 0.3
CUPRAC (mg TE/g)	305.9 ± 6.9
FRAP (mg TE/g)	156.2 ± 2.1
Metal chelating (mg EDTAE/g)	12.7 ± 0.4
Phosphomolybdenum (mmol TE/g)	1.51 ± 0.03
**Enzyme inhibitory properties**	
AChE inhibition (mg GALAE/g)	1.93 ± 0.05
BChE inhibition (mg GALAE/g)	1.14 ± 0.10
Tyrosinase inhibition (mg KAE/g)	46.2 ± 0.2
Amylase inhibition (mmol ACAE/g)	0.45 ± 0.01
Glucosidase inhibition (mmol ACAE/g)	0.11 ± 0.01
Lipase inhibition (mg OE/g)	5.1 ± 0.8

*Note:* Values are reported as mean ± SD of three parallel measurements.

Abbreviations: ACAE, acarbose equivalent; EDTAE, EDTA equivalent; GAE, gallic acid equivalent; GALAE, galanthamine equivalent; KAE, kojic acid equivalent; OE, orlistat equivalent; RE, rutin equivalent; TE, trolox equivalent.

Herein, we evaluated the antioxidant properties of *A. dioica* 80% methanol extract by different chemical methods. The results are presented in Table 2. The radical scavenging activity of the tested extract was evaluated using DPPH and ABTS tests. The *A. dioica* extract demonstrated high radical scavenging activity on DPPH (53.8 ± 0.1 mg TE/g) and ABTS (141.6 ± 0.3 mg TE/g). Reducing power reflects the electron‐donation capacity and assists as an indicator of antioxidant qualities. Accordingly, CUPRAC and FRAP assays were made including the transformation of Cu^2+^ to Cu^+^ and Fe^3+^ to Fe^2+^, respectively. PBD test is similarly considered as a total antioxidant analysis, involving the transformation by antioxidants of Mo (VI) to Mo (V). Hydroxyl radical formation in the Fenton reaction regulated by the chelation of transition metals disclosed as a significant antioxidant mechanism. The studied extract demonstrated strong activity in CUPRAC (305.9 ± 6.9 mg TE/g) and FRAPS (156.2 ± 2.1 mg TE/g) assays, as well as metal chelating capacity of 12.7 ± 0.4 mg EDTAE/g. The radical scavenging activity of *A. dioica* has been previously reported (Babotă et al. 2018). The 70% ethanolic extract demonstrated the highest value (15.21 mg TE/mL), followed by the methanol extract (13.44 ± 1.65 mg TE/mL), whereas the absolute ethanolic extract showed a value of 5.89 mg TE/mL. Furthermore, the 70% ethanolic extract exerted the strongest TEAC activity (5.71 ± 0.01 mg TE/mL) (Babotă et al. 2018).

Enzyme inhibition theory has gained interest in recent decades because it targets key enzymes, potentially alleviating symptoms of health problems such as Alzheimer's disease, diabetes, and obesity (Singh et al. 2024). For example, inhibiting AChE can increase the level of acetylcholine in the synaptic gap, enhancing the cognitive function of Alzheimer's patients (Pandya et al. 2026). Similarly, inhibiting amylase and glucosidase can help to control blood glucose levels in diabetic patients after a carbohydrate‐rich meal (Kumar and Kayastha 2026). Several compounds have therefore been synthesized as enzyme inhibitors in the pharmaceutical industry. However, concerns have been raised regarding their use, such as gastrointestinal disturbances or toxicity (D'Souza et al. 2024). Therefore, effective and safe alternatives are needed.

In light of the abovementioned information, we examined the enzyme inhibitory properties of *A. dioica* extract against cholinesterases, tyrosinase, *α*‐amylase, *α*‐glucosidase, and lipase. The results are shown in Table 2. The extract demonstrated moderate AChE and BChE inhibitory potential and very high activity against tyrosinase (46.2 ± 0.2 mg KAE/g) (Table 2). Tyrosinase is responsible for melanin biosynthesis and plays a key role in skin pigmentation. Tyrosinase inhibitors are important as hypopigmenting agents in cosmetic and medicinal products. Concerning enzymes implicated in the metabolism of carbohydrates and lipids, *A. dioica* revealed moderate *α*‐glucosidase, *α*‐amylase, and lipase inhibitory effects. Inhibition of the aforementioned enzymes played an important role in the therapeutic strategy for control of blood glucose levels in patients with diabetes type II and metabolic disorders. In this framework, the *A. dioica* extract could be used as a multidirectional bioactive agent and a valuable source of bioactive compounds.

### 3.3 In Slilico Investigation

To advance the search for bioactive compounds underlying the observed enzyme affinity and to highlight potential hits for later optimization and pharmacological evaluation, docking studies were carried out on four enzymes—AchE, BChE, *α*‐amylase, and *α*‐glucosidase. First, the docking protocol was validated through re‐docking of crystallographic ligands of the complexes extracted from PDB (https://www.rcsb.org/). The results from the re‐docking procedure are shown in Table 3. The docking poses with best docking scores showed RMSD (root‐mean square deviation) values < 1 Å to the crystallographic poses, suggesting good performance of the docking procedure.

**TABLE 3:** Re‐docking results of the crystallographic ligands.

Enzyme (PDB code)	AChE (4EY7)	BChE (7AMZ)	*α*‐amylase (1Z32)	*α*‐glucosidase (5NN6)
Ligand	E20	RNZ	GLC_AGL_HMC	MIC
Docking score – best pose (kcal/mol)	−9.961	−10.187	−8.143	−6.655
RMSD^a^ (Å)	0.205	0.928	0.636	0.793

^a^ RMSD between the best docking pose and the co‐crystallized ligand.

**FIGURE 4:**
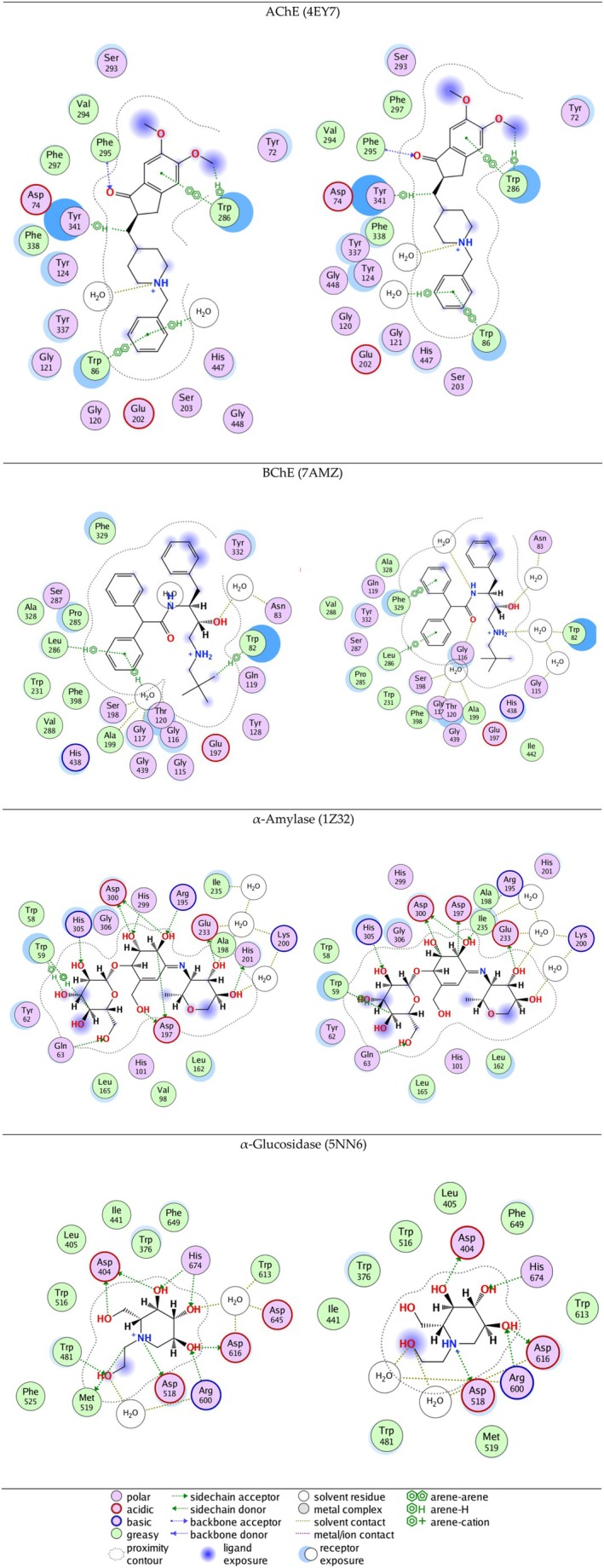
Interactions between the ligands and enzymes for the crystallographic ligands (left panel) and best docking poses after re‐docking (right panel).

The ligands‐enzymes interactions for the crystallographic ligands (left panel) and the best docking poses (right panel) are presented in Figure 4 for comparison. The re‐docked poses and the crystallographic ligands display comparable interactions across all four enzymes, confirming the reliability of the docking protocol.

The docking results for the compounds from *A. dioica* extract having best docking scores are presented in Table 4A (for AChE and BChE) and B (for *α*‐amylase and *α*‐glucosidase). The compounds with the best docking scores are reported. In addition, a cut‐off value set at 0.5 kcal/mol higher than the best score of the corresponding crystallographic ligand (Table 3), was applied; compounds with docking scores better (i.e., lower) than this threshold was also included in Table 4. For each selected compound, the docking scores of all investigated isomers are presented in the Table. For some compounds there were several isomers in the extracts, and therefore they were numbered with different numbers (see Table 1). For compounds with only one isomer found it the extract but not completely defined, docking was done for all positional isomers of this compound, therefore such isomers have one and the same compound number in Table 4.

**TABLE 4:** Docking scores of the selected compounds and their isomers.

A. AChE and BChE docking scores for compounds with the best docking scores and with docking scores better (lower) than the cut‐off values for the both enzymes. The cut‐off values for the docking scores are −9.4 (AChE) and −9.6 (BChE). The docking scores for the positional isomers of the compounds are also presented (separated with lines). The compounds with best (lowest) docking scores for the enzymes, are indicated			
Compound number	Isomer	AChE	BChE
44	2,3‐dicaffeoylglucaric acid	−8.265	−8.641
45	2,4‐dicaffeoylglucaric acid	−8.397	−8.864
46	2,5‐dicaffeoylglucaric acid	−9.527	−8.742
47	3,4‐dicaffeoylglucaric acid	−8.939	−9.082
48 (AchE best score)	3,5‐dicaffeoylglucaric acid	−9.619	−8.384
49	4,5‐dicaffeoylglucaric acid	−8.586	−8.672
50	2,3,4‐tricaffeoylglucaric acid‐*β*‐glucoside	−9.484	−10.293
51	2,3,4‐tricaffeoylglucaric acid	−9.232	−9.995
52	2,4,5‐tricaffeoylglucaric acid	−9.403	−9.987
53	2,3,5‐tricaffeoylglucaric acid	−8.440	−8.237
53	3,4,5‐tricaffeoylglucaric acid	−8.109	−9.475
62	2‐butanyl‐3,4,5‐tricaffeoylglucaric acid	−8.788	−10.419
62	3‐butanyl‐2,4,5‐tricaffeoylglucaric acid	−8.461	−10.640
62 (BChE best score)	4‐butanyl‐2,3,5‐tricaffeoylglucaric acid	−9.071	−11.668
62	5‐butanyl‐2,3,4‐tricaffeoylglucaric acid	−7.757	−7.757

B. *α*‐amylase and *α*‐glucosidase docking scores for compounds with the best docking scores and with docking scores better (lower) than the cut‐off values for the both enzymes. The cut‐off values for the docking scores are −7.6 (*α*‐amylase) and −6.1 (*α*‐glucosidase). The docking scores for the positional isomers of the compounds are also presented (separated with lines). The compounds with best (lowest) docking scores for the enzymes, are indicated			
Compound number	Isomer	*α*‐amylase	*α*‐glucosidase
13	3‐caffeoyl‐5‐ (2‐hydroxy‐dihydrocaffeoyl)quinic acid	−8.477	−6.385
14	3‐caffeoyl‐4‐ (2‐hydroxy‐dihydrocaffeoyl)quinic acid	−8.544	−6.521
16	1‐caffeoyl‐3‐(2‐hydroxydihydrocaffeoyl)quinic acid	−8.364	−6.363
19	1, 4‐dicaffeoylquinic acid	−7.729	−6.148
21	3,4‐dicaffeoylquinic acid	−8.577	−6.585
22	3,5‐dicaffeoylquinic acid	−8.156	−6.613
23	1,5‐dicaffeoylquinic acid	−7.807	−6.831
24	4,5‐trans‐dicaffeoylquinic acid	−7.796	−6.743
25	1‐(*p*‐coumaroyl)‐4‐caffeoylquinic acid	−8.120	−6.441
26	3‐caffeoyl‐4‐ (*p*‐coumaroyl)quinic acid	−7.809	−6.815
27	3‐feruloyl‐4‐caffeoylquinic acid	−8.289	−7.227
28	3‐(*p*‐coumaroyl)‐5‐caffeylquinic acid	−7.736	−6.329
29	3‐trans‐caffeyol‐5‐(*p*‐coumaroyl)quinic acid	−8.216	−6.691
31	3‐cys‐caffeyl‐5‐(*p*‐coumaroyl)quinic acid	−8.318	−6.793
31	1‐(*p*‐coumaroyl)‐5‐caffeylquinic acid	−7.671	−6.702
32	3‐feruloyl‐5‐caffeoylquinic acid	−8.383	−6.856
33	3‐caffeoyl‐5‐feruloylquinic acid	−8.593	−6.767
34	4, 5‐cys‐dicaffeoylquinic acid	−8.320	−6.782
35	4‐(*p*‐coumaroyl)‐5‐caffeylquinic acid	−7.755	−6.670
36	4‐feruloyl‐5‐caffeoylquinic acid	−8.030	−7.236
37	4‐caffeyl‐5‐feruloylquinic acid	−8.062	−6.762
38	4‐caffeyl‐5‐(*p*‐coumaroyl)quinic acid	−7.937	−6.911
39	3,4,5‐tricaffeoylquinic acid	−7.717	−7.011
44	2,3‐dicaffeoylglucaric acid	−8.853	−6.711
45	2,4‐dicaffeoylglucaric acid	−8.138	−6.861
46	2,5‐dicaffeoylglucaric acid	−8.539	−6.245
47	3,4‐dicaffeoylglucaric acid	−7.736	−6.867
48	3,5‐dicaffeoylglucaric acid	−8.319	−7.149
49	4,5‐dicaffeoylglucaric acid	−8.245	−6.946
50	2,3,4‐tricaffeoylglucaric acid‐*β*‐glucoside	−8.238	−6.947
51	2,3,4‐tricaffeoylglucaric acid	−8.281	−7.327
52	2,4,5‐tricaffeoylglucaric acid	−7.674	−7.463
53	2,3,5‐tricaffeoylglucaric acid	−7.893	−7.085
53	3,4,5‐tricaffeoylglucaric acid	−7.958	−7.576
54	2‐(3‐hydroxybutanyl)‐3,4,5‐tricaffeoylglucaric acid (leontopodic acid A)	−8.180	−7.326
55	3‐(3‐hydroxybutanyl)‐2,4,5‐tricaffeoylglucaric acid	−8.094	−6.793
56	4‐(3‐hydroxybutanyl)‐2,3,5‐tricaffeoylglucaric acid	−8.375	−7.796
58	5‐(3‐hydroxybutanyl)‐2,3,4‐tricaffeoylglucaric acid	−8.588	−7.7105
57	2‐(3‐hydroxyisovaleryl)‐ 3,4,5‐tricaffeoylglucaric acid	−8.606	−7.936
57	2‐(4‐hydroxyvaleryl)‐ 3,4,5‐tricaffeoylglucaric acid	−8.264	−7.626
57	2‐(3‐hydroxy‐2‐metilbutiryl)‐3,4,5‐tricaffeoylglucaric acid	−8.716	−7.124
59	3‐(3‐hydroxyisovaleryl)‐ 2,4,5‐tricaffeoylglucaric acid	−8.861	−7.175
59	3‐(4‐hydroxyvaleryl)‐ 2,4,5‐tricaffeoylglucaric acid	−8.647	−6.734
59	3‐(3‐hydroxy‐2‐metilbutiryl)‐2,4,5‐tricaffeoylglucaric acid	−8.098	−7.817
59	4‐(3‐hydroxyisovaleryl)‐ 2,3,5‐tricaffeoylglucaric acid	−8.091	−7.346
59	4‐(4‐hydroxyvaleryl)‐ 2,3,5‐tricaffeoylglucaric acid	−8.826	−7.860
59	4‐(3‐hydroxy‐2‐metilbutiryl)‐2,3,5‐tricaffeoylglucaric acid	−9.276	−7.810
59	5‐(3‐hydroxyisovaleryl)‐ 2,3,4‐tricaffeoylglucaric acid	−8.587	−7.566
59	5‐(4‐hydroxyvaleryl)‐ 2,3,4‐tricaffeoylglucaric acid	−9.191	−7.178
59	5‐(3‐hydroxy‐2‐metilbutiryl)‐2,3,4‐tricaffeoylglucaric acid	−9.631	−7.176
60	2,3,4,5‐trans‐tertracaffeoylglucaric acid	−8.352	−7.377
61	2,3,4,5cys‐tertracaffeoylglucaric acid	−8.766	−6.400
62	2‐butanyl, 3,4,5‐tricaffeoylglucaric acid	−9.032	−7.678
62	3‐butanyl‐2,4,5‐tricaffeoylglucaric acid	−8.767	−7.631
62	4‐butanyl‐2,3,5‐tricaffeoylglucaric acid	−8.737	−7.113
62	5‐butanyl‐2,3,4‐tricaffeoylglucaric acid	−7.819	−6.955
63	2‐isobutanyl‐3,4,5‐tricaffeoylglucaric acid	−8.438	−6.979
63	3‐isobutanyl‐2,4,5‐tricaffeoylglucaric acid	−8.260	−6.996
63	4‐isobutanyl‐2,3,5‐ tricaffeoylglucaric acid	−7.676	−7.071
63	5‐isobutanyl‐2,3,4‐tricaffeoylglucaric acid	−8.647	−7.153
64	2‐(2‐methylbutyl)‐3,4,5‐tricaffeoylglucaric acid	−8.292	−8.117
64	3‐(2‐methylbutyl)‐2,4,5‐tricaffeoylglucaric acid	−8.281	−7.665
64	4‐(2‐methylbutyl)‐2,3,5‐tricaffeoylglucaric acid	−8.403	−7.3121
64 (*α*‐amylase best score)	5‐(2‐methylbutyl)‐2,3,4‐tricaffeoylglucaric acid	−9.822	−7.346
64	2‐isovaleryl‐3,4,5‐tricaffeoylglucaric acid	−8.337	−7.037
64	3‐isovaleryl‐2,4,5‐tricaffeoylglucaric acid	−7.643	−7.499
64	4‐isovaleryl‐2,3,5‐tricaffeoylglucaric acid	−7.933	−7.516
64	5‐isovaleryl‐2,3,4‐tricaffeoylglucaric acid	−8.089	−7.156
65	2‐(p‐hydroxybensoyl)‐3,4,5‐tricaffeoylglucaric acid	−8.478	−7.783
65 (*α*‐glucosidase best score)	3‐(p‐hydroxybensoyl)‐ 2,4,5‐tricaffeoylglucaric acid	−8.993	−8.149
65	4‐(p‐hydroxybensoyl)‐2,3,5‐tricaffeoylglucaric acid	−9.556	−7.445
65	5‐(p‐hydroxybensoyl)‐ 2,3,4‐tricaffeoylglucaric acid	−8.541	−7.453
103	Rutin	−7.874	−6.468
105	Patuletin 3‐*O*‐rutinoside	−7.918	−6.874
107	7‐(6‐hydroxyluteolin)‐malonyl‐*β*‐glucoside	−7.797	−6.453
112	Isorhamnetin 3‐*O*‐rutinoside	−8.099	−6.431
119	Luteolin 7‐*O*‐pentosyl‐*β*‐glucoside	−8.215	−6.549
125	Apigenin 7‐*O*‐caffeoyl‐*β*‐glucoside	−8.025	−6.907

A larger number of compounds displayed favorable docking scores for *α*‐amylase and *α*‐glucosidase than for AChE and BChE, consistent with the plant's use in traditional medicine. Notably, the isomers of the top‐scoring compounds also exhibited similarly good docking scores. Thus, as the extracts contain different isomers of the compounds, the effect of the extracts may be due to the presence of different compounds/isomers with good binding affinities to the enzymes, and not only to a single compound.

Assessment of pharmacokinetic properties and toxicity is essential for selecting viable drug candidates; therefore, next the ADME/Tox (Absorption, Distribution, Metabolism, Excretion, and Toxicity) properties of the compounds were evaluated in silico. Early evaluation of these parameters is critical because many drug candidates fail in later stages of development due to unfavorable pharmacokinetic behavior or unacceptable toxicity profiles. Computational ADME/Tox predictions enable rapid screening of large numbers of compounds, allowing researchers to prioritize molecules with more favorable pharmacokinetic characteristics before conducting costly experimental studies. Despite this, the in silico ADME/Tox evaluations have several limitations related to the reliability of the data used for prediction model development, the model applicability domain, the complexity of the toxicity effects, etc. However, in the last decades, model development was aimed at overcoming the above limitations by using reliable data, rigorous validation procedures, and effective determination of model applicability. As a result, in silico ADME/Tox evaluation has become very important in the early stages of drug development to optimize drug candidates and to reduce costs and time (Shinde et al. 2025). Using different software for toxicity prediction can improve confidence in the results through a consensus‐based approach. Different software relies on distinct algorithms, datasets, and predictive methodologies, which may produce varying outputs for the same compound. Thus, combining predictions from complementary tools can help reduce the risk of unreliable predictions. Therefore, we used both ACD Labs/Percepta software and Derek Nexus expert system.

For all compounds listed in Table 4B, the HIA percentages were < 5%, except for compound 125. Therefore, it was chosen for further investigation in relation to *α*‐amylase and *α*‐glucosidase enzymes.

The molecular and ADME/Tox properties as calculated in ACD Labs/Percepta software and Derek Nexus expert system are presented in Table 5. The compounds with the best docking pose for the enzymes (48, 62, 64, 65), the compounds with good docking scores common for AChE and BChE (50, 52), and compound 125 chosen in relation to *α*‐amylase and *α*‐glucosidase affinity and having good HIA are reported. The properties are the same for the different positional isomers.

**TABLE 5:** Molecular and ADME/Tox properties of the selected compounds.

Compound number	48	50	52	62	64	65	125
A. Properties calculated with ACD Labs/Percepta (Reliability Index (RI) values are presented in brackets)							
MW	534	859	696	767	781	816	595
log*p* (RI)	0.08 (0.60)	−1.42 (0.50)	0.90 (0.64)	2.18 (0.66)	2.4 (0.65)	2.16 (0.59)	1.60 (0.65)
HIA (%)	0	0	0	0	0	0	86
Probability of positive Ames test (RI)	0.02 (0.53)	0.04 (0.48)	0.02 (0.58)	0.02 (0.53)	0.01 (0.52)	0.01 (0.50)	0.29 (0.20)
hERG Inhibitor (Ki < 10 μM) probability (RI)	0.00 (0.31)	0.00 (0.31)	0.00 (0.30)	0.00 (0.20)	0.00 (0.20)	0.00 (0.31)	0.01 (0.38)
B. Alert counts calculated with Derek Nexus for mammals. In the cases where the alert count was 1 the level of likelihood was plausible							
Hepatotoxicity	0	0	0	0	0	0	0
Skin irritation/corrosion	1	1	1	1	1	1	1
Skin sensitization	1	1	1	1	1	1	1
Irritation (of the gastrointestinal tract)	0	0	0	0	1	0	0
Carcinogenicity mammal	0	0	0	0	0	0	0
Nephrotoxicity	0	0	0	0	0	0	0
Androgen receptor modulation	0	0	0	0	0	0	0
Photoallergenicity	0	0	0	0	0	0	0
Estrogen receptor modulation	0	0	0	0	0	0	0

In addition to the properties presented in Table 5A, the blood–brain barrier (BBB) penetration potential and the probability for interaction with Pgp were calculated. The compounds were predicted not to penetrate BBB and not to interact with Pgp.

The compounds have moderate values of log*p* (between −1.42 and 2.18) favorable for drug development. They are predicted to have low toxicity (Table 5), possessing alerts only for skin irritation/corrosion and skin sensitization, except compound 64 which may also cause irritation of the gastrointestinal tract. A positive Ames test indicates a potential for causing mutations in bacterial DNA, thus suggesting possible mutagenicity and carcinogenicity of the compound (Mortelmans and Zeiger 2000). All compounds have low probability for positive Ames test (Table 5A); however, for compound 125 the RI is below 0.3 indicating uncertainty in the prediction. hERG inhibition is an indication for cardiotoxicity (Sanguinetti and Tristani‐Firouzi 2006). The compounds are predicted to have zero probability for being hERG inhibitors.

For compounds **50** and **52** (Table 5B), which have docking scores for AChE and BChE better than the cut‐off values, no other alerts except skin effects were outlined. Although these compounds exhibit poor predicted passive absorption, this is not a significant limitation because many drugs for neurodegenerative diseases are administered parenterally. Compound **52** has a lower molecular weight and a higher log*p* value suggesting better potential for being a lead compound.

**FIGURE 5:**
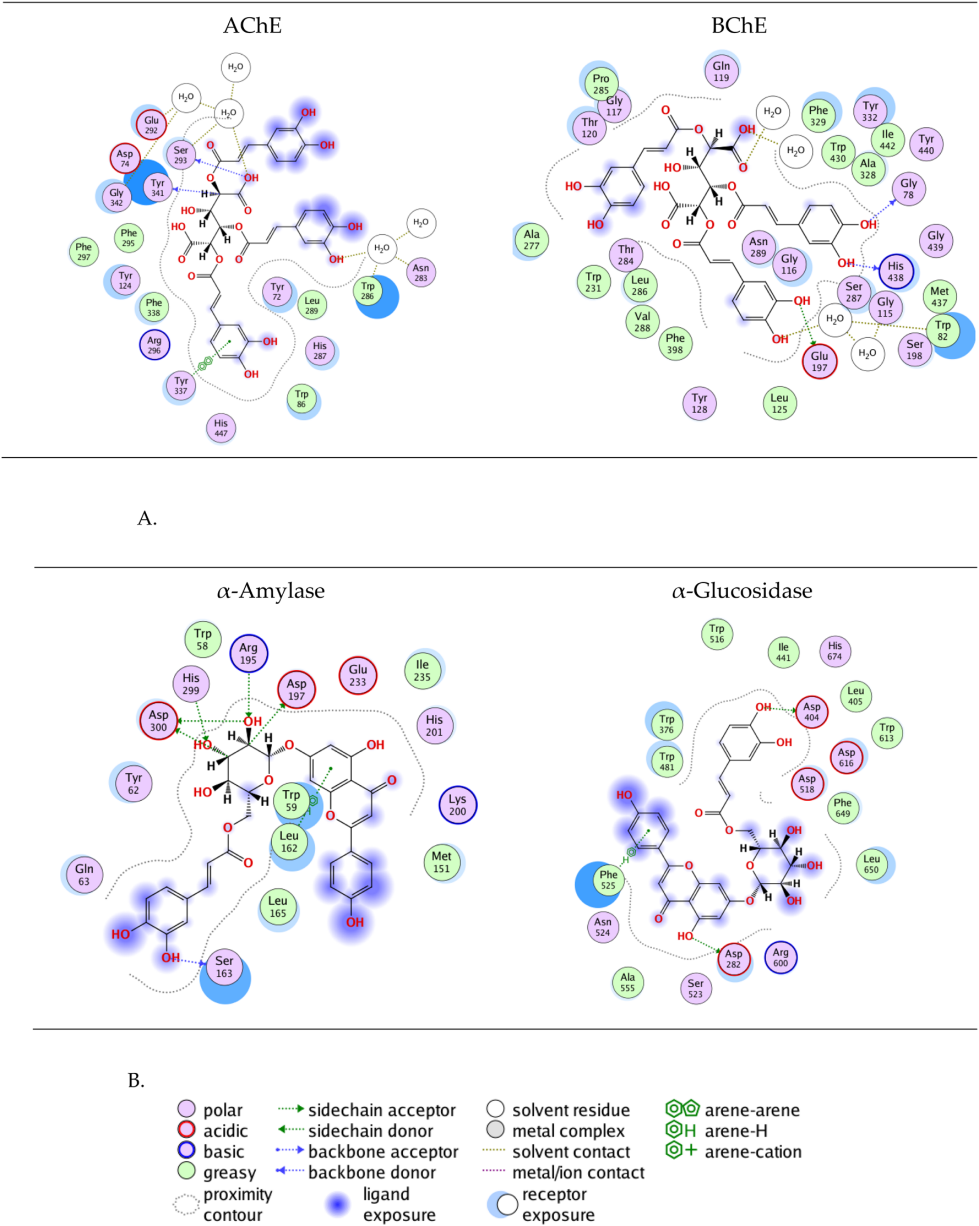
Protein‐ligand interactions of compounds **52** and **125**. (A) Interactions of compound 52 in the binding sites of AChE and BChE. (B) Interactions of compound 125 in the binding sites of *α*‐amylase and *α*‐glucosidase.

The interactions of compound **52** in the binding sites of AChE and BChE are presented in Figure 5A. For AChE, interactions with Trp286 and Tyr341 were observed for both the crystallographic ligand and compound **52**. The investigated compound also exhibited interaction with Ser293 (H‐bond) and Asn283, Glu292, Tyr337, and Glu342 through water bridges. For BChE H‐bonds with Gly78, Glu197, and His438 were observed, and interaction with Trp82 was shown in both the crystallographic ligand and compound 52. H‐bonds between ligands and Tyr337, Ser293 in AChE, and Glu197 in BChE are also shown in other docking studies (Islam et al. 2013; Mascarenhas et al. 2021; Ul‐Haq et al. 2010).

Apigenin 7‐*O*‐caffeoyl‐*β*‐glucoside (compound **125**) was outlined as having good docking scores in *α*‐amylase and *α*‐glucosidase, high predicted intestinal absorption and in general a favorable toxicity profile. Its interactions in the binding sites of *α*‐amylase and *α*‐glucosidase are presented in Figure 5B. In *α*‐amylase interactions with Trp59, Arg195, Asp197, Asp300 were observed for both compound 125 and the crystallographic ligand. Such ligand interactions with *α*‐amylase are reported also in the literature (Aminu et al. 2024; Anigboro et al. 2021; Ibrahim et al. 2019). The investigated compound interacts with Asp282, Asp404 (present also in the interaction of the crystallographic ligand), Phe525 in the site of *α*‐glucosidase, in accordance with literature reports (Bashyal et al. 2024).

The molecular docking study and the in vitro enzyme inhibition assays address different but complementary aspects of the biological activity of the extracts. While the in vitro experiments evaluate the overall inhibitory activity of the extracts against the tested enzymes, the molecular docking analysis investigates the ability of individual phytochemical constituents identified in the extracts to interact with the active sites of the target enzymes at the molecular level. According to the docking results, the extracts contain compounds with good potential to bind to AChE, BChE, *α*‐amylase, and *α*‐glucosidase, which is consistent with the experimentally observed moderate inhibitory activity of the extracts against these enzymes. In addition, the docking studies reveal the molecular mechanisms of the compound‐enzyme interactions. Such interactions may contribute to the inhibitory effects observed experimentally and help rationalize the measured enzyme inhibition.

Nevertheless, it should be emphasized that the in silico results represent a predictive computational approach and cannot directly substitute experimental validation. Therefore, the in silico findings, including docking results and toxicity predictions, should be interpreted as supportive evidence that helps explain the observed biological activity and guides future research.

## 4 Conclusions

In line with the new paradigm in pharmacognosy to obtain in‐depth metabolite profiling of natural extracts for a rational prioritization of bioactive natural compounds, this study was designed to investigate the phytochemical profile and biological activity of *A. dioica* extract. Herein, a total of 130 secondary metabolites, including acylquinic, caffeoylhexaric, carboxylic, phenolic acids, coumarins, and flavonoids were dereplicated/annotated by means of UHPLC‐HRMS/MS. Taken together, our data suggest more than 68 core structures reported in the species for the first time. *A. dioica* provides a rich source of CHAs and unique nature of its profiling could be associated with the leontopodic acids A and B. The percentage ratio of the assayed classes revealed the highest relative content of AQAs (55.62%), followed by hydroxybenzoic and hydroxycinnamic acids and derivatives (21.70%), CHAs (15.79%), and flavonoids (6.85%). Chlorogenic, 3,5‐dicaffeoylquinic, neochlorogenic, leontopodic acids A and B, protocatechuic acid *O*‐hexoside, myricetin *O*‐hexoside, luteolin 7‐*O*‐glucoside and luteolin dominated in the extract profiling. The pronounced antioxidant activity (DDPH, FRAP, CUPRAC, ABTS, Chelating, and Phosphomolibdenum capacity) and enzyme inhibitory potential towards AChE, BChE, tyrosinase, and lipase of the extract was observed.

The compounds identified in the extract of *A. dioica* were investigated with in silico approaches for their potential to bind AChE and BChE, as well as *α*‐amylase and *α*‐glucosidase. Their ADME/Tox properties were also evaluated. The extract was found to contain more potential ligands of *α*‐amylase and *α*‐glucosidase than of AChE and BChE in accordance with the use of the plant in traditional medicine. Numerous isomers with favorable docking scores were detected, suggesting that the observed effects may be due to the presence of different compounds. Compounds predicted to be active ligands of AChE and BChE have favorable toxicological profiles, but poor intestinal absorption due to their low log*p* values. Further optimization of these hit molecules may include introducing substitutes which increase their lipophilicity, and thus their passive absorption. Among the compounds, apigenin 7‐*O*‐caffeoyl‐*β*‐glucoside was outlined as having good potential for interaction with *α*‐amylase and *α*‐glucosidase, and favorable ADME/Tox properties. We demonstrated that *A. dioica* aerial parts extract may reduce oxidative stress and have beneficial effects on metabolic disorders and skin imperfections.

## Author Contributions

**Dimitrina Zheleva‐Dimitrova:** conceptualization, software, resources, data curation, writing – original draft, visualization, supervision, methodology. **Ivanka Tsakovska:** conceptualization, methodology, validation, resources, writing – original draft, supervision. **Reneta Gevrenova:** methodology, software, writing – review and editing, writing – original draft. **Gokhan Zengin:** methodology, writing – original draft, formal analysis, data curation. **Ivayla Zheleva‐Kyuchukova:** resources, writing – original draft. **Radostina Nikolova‐Kejova:** investigation, data curation. **Iglika Lessigiarska:** conceptualization, methodology, validation, formal analysis, data curation, writing – original draft, writing – review and editing. **Georgi Momekov:** funding acquisition, project administration.

## Funding

This research is financed by the European Union—NextGenerationEU, through the National Recovery and Resilience Plan of the Republic of Bulgaria, project BG‐RRP‐2.004‐0004‐C01 “Strategic research and innovation program for development of Medical University—Sofia.”

## Supporting information


**Figure S1:** Extracted ion chromatogram (EIC) of acylquinic acids (AQAs). EIC was proceed with mass tolerance of 5 ppm as follows: 1–3 at m/z 371.0984 (371.0965–371.1003); 4, 5, 7, 9 and 11 at m/z 353.0867 (353.0849–353.0885); 6, 12 and 17 at m/z 337.0928 (337.0911–337.0945); 8 at m/z 355.1035 (355.1017–355.1053); 10, 15, 18 and 20 at m/z 367.1034 (367.1016–367.1052); 13, 14 and 16 at m/z 533.1288 (533.1261–533.1315); 19, 21–24 and 34 at m/z 515.1189 (515.1163–515.1215); 25, 26, 28, 29, 31, 35 and 38 at m/z 499.1251 (499.1226–499.1276); 27, 30, 32, 33, 36 and 37 at m/z 529.1356 (529.1330–529.1382); 39 at m/z 677.1512 (677.1478–677.1546) (for numbers and fragmentation patterns, see Table 1).
**Figure S2:** Extracted ion chromatogram at *m/z* 353.0867 (353.0849–353.0885) (mass accuracy 5 ppm) at *t*_R_ 3.19 min. [M‐H]^−^ at 353.0880 and [2 M‐H]^−^ at 707.1831.
**Figure S3:** (−) ESI‐MS/MS spectrum of chlorogenic acid (**7**) at *m/z* 353.0867 (353.0849–353.0885) (mass accuracy 5 ppm) (for numbers and fragmentation patterns, see Table 1).
**Figure S4:** (−) ESI‐MS/MS spectrum of neochlorogenic acid (**4**) at *m/z* 353.0867 (353.0849–353.0885) (mass accuracy 5 ppm) (for numbers and fragmentation patterns, see Table 1).
**Figure S5:** Extracted ion chromatogram at *m/z* 515.1189 (515.1163–515.1215) (mass accuracy 5 ppm) at *t*_R_ 5.84 min. [M‐H]^−^ at 515.1191 and [2 M‐H]^−^ at *m/z* 1031.2462.
**Figure S6:** (−) ESI‐MS/MS spectrum of 1, 5‐dicaffeoylquinic acid (**23**) at *m/z* 515.1189 (515.1163–515.1215) (mass accuracy 5 ppm) (for numbers and fragmentation patterns, see Table 1).
**Figure S7:** (−) ESI‐MS/MS spectrum of 3, 4‐dicaffeoylquinic acid (**21**) at *m/z* 515.1189 (515.1163–515.1215) (mass accuracy 5 ppm) (for numbers and fragmentation patterns, see Table 1).
**Figure S8:** (−) ESI‐MS/MS spectrum of 4, 5‐dicaffeoylquinic acid (**24**) at *m/z* 515.1189 (515.1163–515.1215) (mass accuracy 5 ppm) (for numbers and fragmentation patterns, see Table 1).
**Figure S9:** (−) ESI‐MS/MS spectrum of 3‐caffeoyl‐4‐*p*‐coumaroylquinic acid (**26**) at *m/z* 499.1251 (499.1226–499.1276) (mass accuracy 5 ppm) (for numbers and fragmentation patterns, see Table 1).
**Figure S10:** (−) ESI‐MS/MS spectrum of 4‐caffeoyl‐5‐feruloylquinic acid (**37**) at *m/z* 529.1356 (529.1356–529.1382) (mass accuracy 5 ppm) (for numbers and fragmentation patterns, see Table 1).
**Figure S11:** (−) ESI‐MS/MS spectrum of 3, 4, 5‐tricaffeoylquinic acid (**39**) at *m/z* 677.1512 (677.1448–677.1546) (mass accuracy 5 ppm) (for numbers and fragmentation patterns, see Table 1).
**Figure S12:** Extracted ion chromatogram (EIC) of acylhexaric acids (AHAs). EIC was proceed with mass tolerance of 5 ppm as follows: **40**–**43** at *m/z* 371.0620 (371.0601–371.0639); 4**4**–**49** at *m/z* 533.0937 (533.0910–533.0964); **50** at *m/z* 857.1782 (857.1739–857.1825); **51–53** at *m/z* 695.1254 (695.1219–695.1289); **54–56** and **58** at *m/z* 781.1622 (781.1583–781.1661); **57** and **59** at *m/z* 795.1778 (795.1738–795.1818); **60** and **61** at *m/z* 857.1571 (857.1528–857.1614); **62** and **63** at *m/z* 765.1672 (765.1634–765.1710); **64** at *m/z* 779.1829 (779.1790–779.1868); **65** at *m/z* 815.1465 (815.1424–815.1506) (for numbers and fragmentation patterns, see Table 1).
**Figure S13:** (−) ESI‐MS/MS spectrum of caffeoylhexaric acid (**40**) at *m/z* 371.0620 (371.0601–371.0639) (mass accuracy 5 ppm) (for numbers and fragmentation patterns, see Table 1).
**Figure S14:** (−) ESI‐MS/MS spectrum of dicaffeoylhexaric acid (**44**) at *m/z* 533.0937 (533.0910–533.0964) (mass accuracy 5 ppm) (for numbers and fragmentation patterns, see Table 1).
**Figure S15:** (−) ESI‐MS/MS spectrum of Leontopodic acid B (**51**) at *m/z* 695.1254 (695.1219–695.1289) (mass accuracy 5 ppm) (for numbers and fragmentation patterns, see Table 1).
**Figure S16:** (−) ESI‐MS/MS spectrum of tetracaffeoylhexaric acid (**60**) at *m/z* 857.1571 (857.1528–857.1614) (mass accuracy 5 ppm) (for numbers and fragmentation patterns, see Table 1).
**Figure S17:** (−) ESI‐MS/MS spectrum of hydroxyvaleryl/hydroxyisovaleryl‐tricaffeoylhexaric acid (**57**) at *m/z* 795.1778 (795.1738–795.1818) (mass accuracy 5 ppm) (for numbers and fragmentation patterns, see Table 1).
**Figure S18:** (−) ESI‐MS/MS spectrum of butanyl/isobutanyl‐tricaffeoylhexaric acid (**62**) at *m/z* 765.1672 (765.1634–765.1710) (mass accuracy 5 ppm) (for numbers and fragmentation patterns, see Table 1).
**Figure S19:** Extracted ion chromatogram (EIC) of carboxylic and phenolic acids, coumarins, and derivatives. EIC was proceed with mass tolerance of 5 ppm (see below) (for numbers and fragmentation patterns, see Table 1).
**Figure S20:** (−) ESI‐MS/MS spectrum of mallic acid (**66**) at *m/z* 133.0142 (for numbers and fragmentation patterns, see Table 1).
**Figure S21:** (−) ESI‐MS/MS spectrum of protocatechuic acid (**70**) at *m/z* 153.0181 (153.0173–153.0189) (mass tolerance 5 ppm) (for numbers and fragmentation patterns, see Table 1).
**Figure S22:** (−) ESI‐MS/MS spectrum of 4‐hydroxybenzoic acid (**80**) at *m/z* 137.0230 (137.0223–137.0237) (mass tolerance 5 ppm) (for numbers and fragmentation patterns, see Table 1).
**Figure S23:** (−) ESI‐MS/MS spectrum of 3‐hydroxybenzoic acid (**81**) at *m/z* 137.0230 (137.0223–137.0237) (mass tolerance 5 ppm) (for numbers and fragmentation patterns, see Table 1).
**Figure S24:** (−) ESI‐MS/MS spectrum of gentisic acid (**82**) at *m/z* 153.0181 (153.0173–153.0189) (mass tolerance 5 ppm) (for numbers and fragmentation patterns, see Table 1).**Figure S25**. (−) ESI‐MS/MS spectrum of *p*‐coumaric acid (**89**) at *m/z* 163.0389 (163.0381–163.0397) (mass tolerance 5 ppm) (for numbers and fragmentation patterns, see Table 1).
**Figure S26:** (−) ESI‐MS/MS spectrum of *p*‐hydroxyphenylacetic acid (**91**) at *m/z* 151.0401 (151.0393–151.0409) (mass tolerance 5 ppm) (for numbers and fragmentation patterns, see Table 1).
**Figure S27:** (−) ESI‐MS/MS spectrum of caffeic acid (**92**) at *m/z* 179.0339 (179.0330–179.0348) (mass tolerance 5 ppm) (for numbers and fragmentation patterns, see Table 1).
**Figure S28:** (−) ESI‐MS/MS spectrum of *m*‐coumaric acid (**95**) at *m/z* 163.0389 (163.0381–163.0397) (mass tolerance 5 ppm) (for numbers and fragmentation patterns, see Table 1).
**Figure S29:** (−) ESI‐MS/MS spectrum of vanillic acid (**96**) at *m/z* 167.0338 (167.0330–167.0346) (mass tolerance 5 ppm) (for numbers and fragmentation patterns, see Table 1).
**Figure S30:** (−) ESI‐MS/MS spectrum of *o*‐coumaric acid (**97**) at *m/z* 163.0389 (163.0381–163.0397) (mass tolerance 5 ppm) (for numbers and fragmentation patterns, see Table 1).
**Figure S31:** (−) ESI‐MS/MS spectrum of scopoletin (**98**) at *m/z* 191.0350 (191.0340–191.0360) (mass tolerance 5 ppm) (for numbers and fragmentation patterns, see Table 1).
**Figure S32:** (−) ESI‐MS/MS spectrum of salicylic acid (**99**) at *m/z* 137.0230 (137.0223–137.0237) (mass tolerance 5 ppm) (for numbers and fragmentation patterns, see Table 1).
**Figure S33:** Extracted ion chromatogram (EIC) of flavonoids. EIC was proceed with mass tolerance of 5 ppm (for numbers and fragmentation patterns, see Table 1).
**Figure S34:** (−) ESI‐MS/MS spectrum of rutin (**103**) at *m/z* 609.1464 (609.1434–609.1494) (mass accuracy 5 ppm) (for numbers and fragmentation patterns, see Table 1).
**Figure S35:** (−) ESI‐MS/MS spectrum of luteolin 7‐O‐glucoside (**108**) at *m/z* 447.0933 (447.0911–447.0955) (mass accuracy 5 ppm) (for numbers and fragmentation patterns, see Table 1).
**Figure S36:** (−) ESI‐MS/MS spectrum of nepetin 7‐O‐glucoside (**110**) at *m/z* 477.1038 (477.1014–477.1062) (mass accuracy 5 ppm) (for numbers and fragmentation patterns, see Table 1).
**Figure S37:** (−) ESI‐MS/MS spectrum of kaempferol 3‐*O*‐rutinoside (**111**) at *m/z* 593.1512 (593.1014–593.1542) (mass accuracy 5 ppm) (for numbers and fragmentation patterns, see Table 1).**Figure S38**. (−) ESI‐MS/MS spectrum of isorhamnetin 3‐*O*‐rutinoside (**112**) at *m/z* 623.1618 (623.1587–623.1649) (mass accuracy 5 ppm) (for numbers and fragmentation patterns, see Table 1).
**Figure S39:** (−) ESI‐MS/MS spectrum of apigenin 7‐*O*‐glucoside (**112**) at *m/z* 431.0984 (431.0962–431.1006) (mass accuracy 5 ppm) (for numbers and fragmentation patterns, see Table 1).
**Figure S40:** (−) ESI‐MS/MS spectrum of luteolin (**122**) at *m/z* 285.0405 (285.0391–285.0419) (mass accuracy 5 ppm) (for numbers and fragmentation patterns, see Table 1).
**Figure S41:** (−) ESI‐MS/MS spectrum of quercetin (**123**) at *m/z* 301.0354 (301.0339–301.0369) (mass accuracy 5 ppm) (for numbers and fragmentation patterns, see Table 1).
**Figure S42:** (−) ESI‐MS/MS spectrum of nepetin (**124**) at *m/z* 315.0510 (315.0494–315.0526) (mass accuracy 5 ppm) (for numbers and fragmentation patterns, see Table 1).
**Figure S43:** (−) ESI‐MS/MS spectrum of kaempferol (**126**) at *m/z* 285.0405 (285.0391–285.0419) (mass accuracy 5 ppm) (for numbers and fragmentation patterns, see Table 1).
**Figure S44:** (−) ESI‐MS/MS spectrum of apigenin (**127**) at *m/z* 269.0455 (269.0442–269.0468) (mass accuracy 5 ppm) (for numbers and fragmentation patterns, see Table 1).
**Figure S45:** (−) ESI‐MS/MS spectrum of isorhamnetin (**129**) at *m/z* 315.0510 (315.0494–315.0526) (mass accuracy 5 ppm) (for numbers and fragmentation patterns, see Table 1).
**Figure S46:** (−) ESI‐MS/MS spectrum of genkwanin (**130**) at *m/z* 283.0612 (283.0598–283.0626) (mass accuracy 5 ppm) (for numbers and fragmentation patterns, see Table 1).

## Data Availability

The data that supports the findings of this study are available in the Supporting Information of this article.
