# Supplementary material for: Antennaria dioica (L.) Gaertn. (Asteraceae): From Metabolite Profiling to Comprehensive Evaluation of In Vitro and In Silico Enzyme Inhibitory Activity
Source: Food Sci Nutr. 2026 May 13;14(5):e71854. doi: 10.1002/fsn3.71854 (PMC13169136; doi:10.1002/fsn3.71854)
Supplement: Supplementary file 1 — Figure S1: Extracted ion chromatogram (EIC) of acylquinic acids (AQAs). EIC was proceed with mass tolerance of 5 ppm as follows: 1–3 at m/z 371.0984 (371.0965–371.1003); 4, 5, 7, 9 and 11 at m/z 353.0867 (353.0849–353.0885); 6, 12 and 17 at m/z 337.0928 (337.0911–337.0945); 8 at m/z 355.1035 (355.1017–355.1053); 10, 15, 18 and 20 at m/z 367.1034 (367.1016–367.1052); 13, 14 and 16 at m/z 533.1288 (533.1261–533.1315); 19, 21–24 and 34 at m/z 515.1189 (515.1163–515.1215); 25, 26, 28, 29, 31, 35 and 38 at m/z 499.1251 (499.1226–499.1276); 27, 30, 32, 33, 36 and 37 at m/z 529.1356 (529.1330–529.1382); 39 at m/z 677.1512 (677.1478–677.1546) (for numbers and fragmentation patterns, see Table 1). Figure S2: Extracted ion chromatogram at m/z 353.0867 (353.0849–353.0885) (mass accuracy 5 ppm) at t R 3.19 min. [M‐H]− at 353.0880 and [2 M‐H]− at 707.1831. Figure S3: (−) ESI‐MS/MS spectrum of chlorogenic acid (7) at m/z 353.0867 (353.0849–353.0885) (mass accuracy 5 ppm) (for numbers and fragmentation patterns, see Table 1). Figure S4: (−) ESI‐MS/MS spectrum of neochlorogenic acid (4) at m/z 353.0867 (353.0849–353.0885) (mass accuracy 5 ppm) (for numbers and fragmentation patterns, see Table 1). Figure S5: Extracted ion chromatogram at m/z 515.1189 (515.1163–515.1215) (mass accuracy 5 ppm) at t R 5.84 min. [M‐H]− at 515.1191 and [2 M‐H]− at m/z 1031.2462. Figure S6: (−) ESI‐MS/MS spectrum of 1, 5‐dicaffeoylquinic acid (23) at m/z 515.1189 (515.1163–515.1215) (mass accuracy 5 ppm) (for numbers and fragmentation patterns, see Table 1). Figure S7: (−) ESI‐MS/MS spectrum of 3, 4‐dicaffeoylquinic acid (21) at m/z 515.1189 (515.1163–515.1215) (mass accuracy 5 ppm) (for numbers and fragmentation patterns, see Table 1). Figure S8: (−) ESI‐MS/MS spectrum of 4, 5‐dicaffeoylquinic acid (24) at m/z 515.1189 (515.1163–515.1215) (mass accuracy 5 ppm) (for numbers and fragmentation patterns, see Table 1). Figure S9: (−) ESI‐MS/MS spectrum of 3‐caffeoyl‐4‐p‐coumaroylquinic acid (26) at m/z 499.1 [file FSN3-14-e71854-s001.docx]

***Antennaria dioica* (L.) Gaertn. (Asteraceae): from metabolite profiling to comprehensive evaluation of *in vitro* and *in silico* enzyme inhibitory activity**

Dimitrina Zheleva-Dimitrova^1^, Gokhan Zengin^2^, Iglika Lessigiarska^3^, Ivanka Tsakovska^3^, Radostina Nikolova-Kejova^3^, Ivayla Zheleva-Kyuchukova^4^, Georgi Momekov^5^, Reneta Gevrenova^1*^

^1^ Department of Pharmacognosy, Faculty of Pharmacy, Medical University, 1000 Sofia, Bulgaria;

^2^ Physiology and Biochemistry Research Laboratory, Department of Biology, Science Faculty, Selcuk University, Konya 42130, Turkey;

^3^ Institute of Biophysics and Biomedical Engineering, Bulgarian Academy of Sciences, Sofia, Bulgaria

^4^ Acibadem City Clinic UMBAL Tokuda, Sofia, Bulgaria;

^5^ Department of Pharmacology, pharmacotherapy and toxicology, Faculty of Pharmacy, Medical University, 1000 Sofia, Bulgaria;

*Corresponding authors: dzheleva@[pharmfac.mu-sofia.bg](mailto:rgevrenova@pharmfac.mu-sofia.bg);

**Supplemental material**


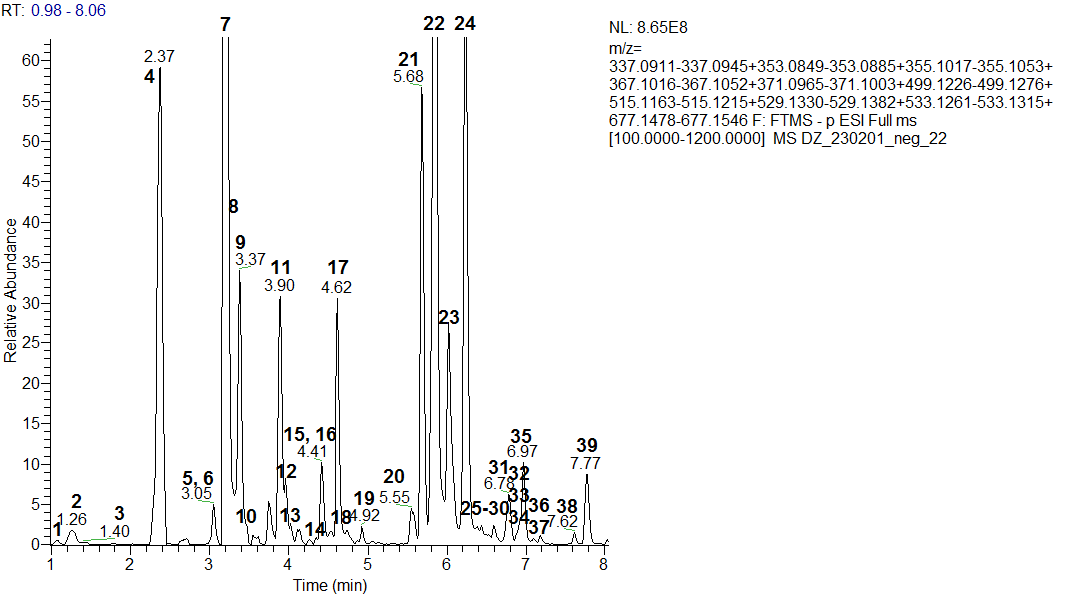
**Figure S1.** Extracted ion chromatogram (EIC)of acylquinic acids (AQAs). EIC was proceed with mass tolerance of 5 ppm as follows: **1**-**3** at *m/z* 371.0984 (371.0965-371.1003); **4**, **5**, **7,** **9** and **11** at *m/z* 353.0867 (353.0849-353.0885); **6**, **12** and **17** at *m/z* 337.0928 (337.0911-337.0945); **8** at *m/z* 355.1035 (355.1017-355.1053); **10**, **15**, **18** and **20** at *m/z* 367.1034 (367.1016-367.1052); **13**, **14** and **16** at *m/z* 533.1288 (533.1261-533.1315); **19**, **21**-**24** and **34** at *m/z* 515.1189 (515.1163-515.1215); **25**, **26**, **28**, **29**, **31,** **35** and **38** at *m/z* 499.1251 (499.1226-499.1276); **27**, **30**, **32**, **33**, **36** and **37** at *m/z* 529.1356 (529.1330-529.1382); **39** at *m/z* 677.1512 (677.1478-677.1546) (for numbers and fragmentation patterns, see Table 1).

**Figure S2.** Extracted ion chromatogram at *m/z* 353.0867 (353.0849-353.0885) (mass accuracy 5 ppm) at t_R_ 3.19 min.[M-H]^-^ at 353.0880 and [2M-H]^-^ at 707.1831.

**Figure S3.** (-) ESI-MS/MS spectrum of chlorogenic acid (7**)** at *m/z* 353.0867 (353.0849-353.0885) (mass accuracy 5 ppm) (for numbers and fragmentation patterns, see Table 1).

**Figure S4.** (-) ESI-MS/MS spectrum of neochlorogenic acid (**4)** at *m/z* 353.0867 (353.0849-353.0885) (mass accuracy 5 ppm) (for numbers and fragmentation patterns, see Table 1).


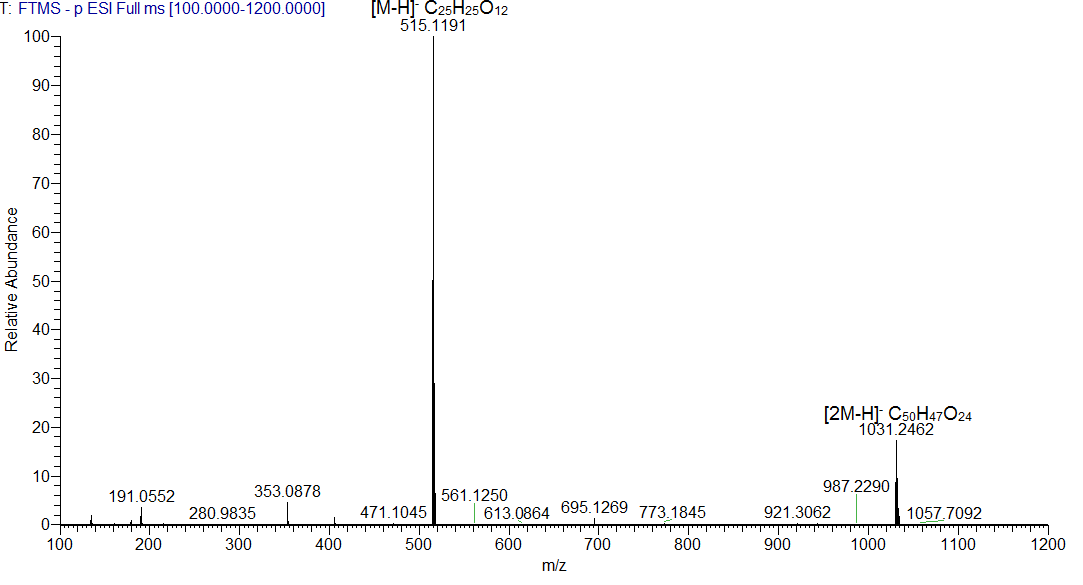


**Figure S5.** Extracted ion chromatogram at *m/z* 515.1189 (515.1163-515.1215) (mass accuracy 5 ppm) at t_R_ 5.84 min.[M-H]^-^ at 515.1191 and [2M-H]^-^ at 1031.2462.

**Figure S6.** (-) ESI-MS/MS spectrum of 3, 4-dicaffeoylquinic acid (**21)** at *m/z* 515.1189 (515.1163-515.1215) (mass accuracy 5 ppm) (for numbers and fragmentation patterns, see Table 1).

**Figure S7.** (-) ESI-MS/MS spectrum of 1, 5-dicaffeoylquinic acid (**23)** at *m/z* 515.1189 (515.1163-515.1215) (mass accuracy 5 ppm) (for numbers and fragmentation patterns, see Table 1).

**Figure S8.** (-) ESI-MS/MS spectrum of 4, 5-dicaffeoylquinic acid (**24)** at *m/z* 515.1189 (515.1163-515.1215) (mass accuracy 5 ppm) (for numbers and fragmentation patterns, see Table 1).

**Figure S9.** (-) ESI-MS/MS spectrum of 3-caffeoyl-4-*p*-coumaroylquinic acid (**26)** at *m/z* 499.1251 (499.1226-499.1276) (mass accuracy 5 ppm) (for numbers and fragmentation patterns, see Table 1).

**Figure S10.** (-) ESI-MS/MS spectrum of 4-caffeoyl-5-feruloylquinic acid (**37)** at *m/z* 529.1356 (529.1356-529.1382) (mass accuracy 5 ppm) (for numbers and fragmentation patterns, see Table 1).

**Figure S11.** (-) ESI-MS/MS spectrum of 3, 4, 5-tricaffeoylquinic acid (**39)** at *m/z* 677.1512 (677.1448-677.1546) (mass accuracy 5 ppm) (for numbers and fragmentation patterns, see Table 1).


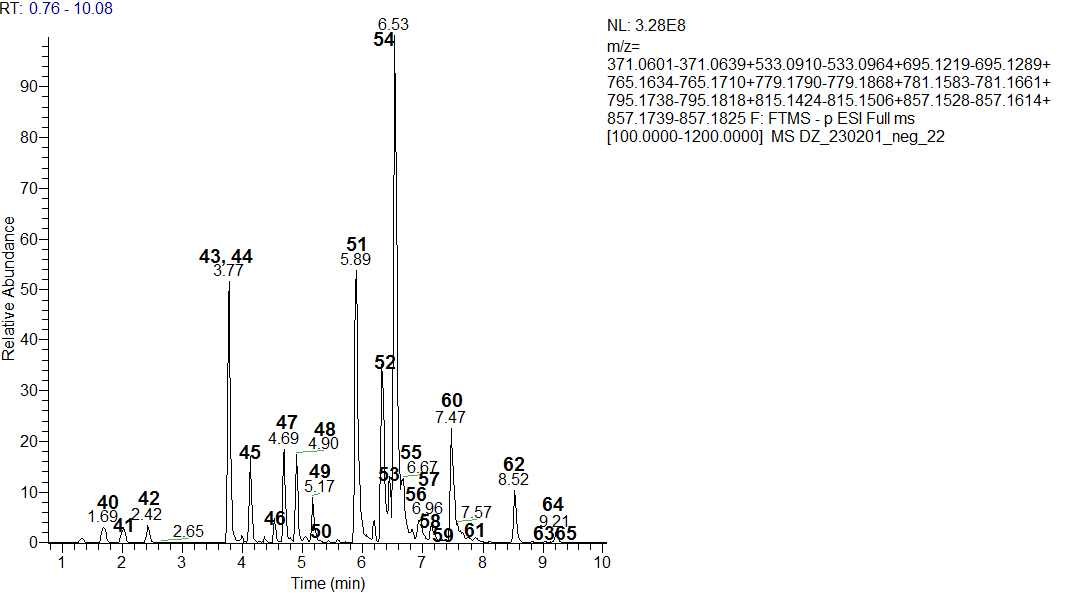


**Figure S12.** Extracted ion chromatogram (EIC)of acylhexaric acids (AHAs). EIC was proceed with mass tolerance of 5 ppm as follows: **40**-**43** at *m/z* 371.0620 (371.0601-371.0639); 4**4**-**49** at *m/z* 533.0937 (533.0910-533.0964); **50** at *m/z* 857.1782 (857.1739-857.1825); **51-53** at *m/z* 695.1254 (695.1219-695.1289); **54-56** and **58** at *m/z* 781.1622 (781.1583-781.1661); **57** and **59** at *m/z* 795.1778 (795.1738-795.1818); **60** and **61** at *m/z* 857.1571 (857.1528-857.1614); **62** and **63** at *m/z* 765.1672 (765.1634-765.1710); **64** at *m/z* 779.1829 (779.1790-779.1868); **65** at *m/z* 815.1465 (815.1424-815.1506) (for numbers and fragmentation patterns, see Table 1).

**Figure S13.** (-) ESI-MS/MS spectrum of caffeoylhexaric acid (**40)** at *m/z* 371.0620 (371.0601-371.0639) (mass accuracy 5 ppm) (for numbers and fragmentation patterns, see Table 1).

**Figure S14.** (-) ESI-MS/MS spectrum of dicaffeoylhexaric acid (**44)** at *m/z* 533.0937 (533.0910-533.0964) (mass accuracy 5 ppm) (for numbers and fragmentation patterns, see Table 1).

**Figure S15.** (-) ESI-MS/MS spectrum of Leontopodic acid B (**51)** at *m/z* 695.1254 (695.1219-695.1289) (mass accuracy 5 ppm) (for numbers and fragmentation patterns, see Table 1).

**Figure S16.** (-) ESI-MS/MS spectrum of tetracaffeoylhexaric acid (**60)** at *m/z* 857.1571 (857.1528-857.1614) (mass accuracy 5 ppm) (for numbers and fragmentation patterns, see Table 1).

**Figure S17.** (-) ESI-MS/MS spectrum of hydroxyvaleryl/hydroxyisovaleryl-tricaffeoylhexaric acid (**57)** at *m/z* 795.1778 (795.1738-795.1818) (mass accuracy 5 ppm) (for numbers and fragmentation patterns, see Table 1).

**Figure S18.** (-) ESI-MS/MS spectrum of butanyl/isobutanyl-tricaffeoylhexaric acid (**62)** at *m/z* 765.1672 (765.1634-765.1710) (mass accuracy 5 ppm) (for numbers and fragmentation patterns, see Table 1).


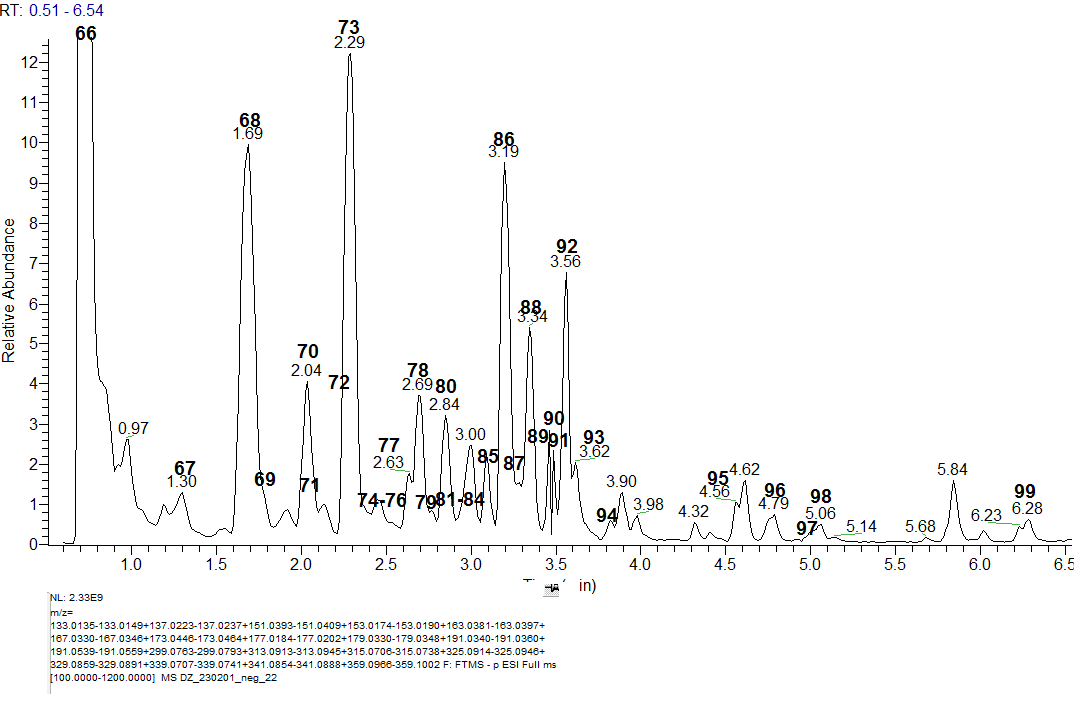


**Figure S19.** Extracted ion chromatogram (EIC)of carboxylic and phenolic acids, coumarins, and derivatives. EIC was proceed with mass tolerance of 5 ppm (see below) (for numbers and fragmentation patterns, see Table 1).

**Figure S20.** (-) ESI-MS/MS spectrum of mallic acid (**66)** at *m/z* 133.0142 (for numbers and fragmentation patterns, see Table 1).

**Figure S21.** (-) ESI-MS/MS spectrum of protocatechuic acid (**70)** at *m/z* 153.0181 (153.0173-153.0189) (mass tolerance 5 ppm) (for numbers and fragmentation patterns, see Table 1).

**Figure S22.** (-) ESI-MS/MS spectrum of 4-hydroxybenzoic acid (**80)** at *m/z* 137.0230 (137.0223-137.0237) (mass tolerance 5 ppm) (for numbers and fragmentation patterns, see Table 1).

**Figure S23.** (-) ESI-MS/MS spectrum of 3-hydroxybenzoic acid (**81)** at *m/z* 137.0230 (137.0223-137.0237) (mass tolerance 5 ppm) (for numbers and fragmentation patterns, see Table 1).

**Figure S24.** (-) ESI-MS/MS spectrum of gentisic acid (**82)** at *m/z* 153.0181 (153.0173-153.0189) (mass tolerance 5 ppm) (for numbers and fragmentation patterns, see Table 1).

**Figure S25.** (-) ESI-MS/MS spectrum of *p*-coumaric acid (**89)** at *m/z* 163.0389 (163.0381-163.0397) (mass tolerance 5 ppm) (for numbers and fragmentation patterns, see Table 1).

**Figure S26.** (-) ESI-MS/MS spectrum of *p*-hydroxyphenylacetic acid (**91)** at *m/z* 151.0401 (151.0393-151.0409) (mass tolerance 5 ppm) (for numbers and fragmentation patterns, see Table 1).

**Figure S27.** (-) ESI-MS/MS spectrum of caffeic acid (**92)** at *m/z* 179.0339 (179.0330-179.0348) (mass tolerance 5 ppm) (for numbers and fragmentation patterns, see Table 1).

**Figure S28.** (-) ESI-MS/MS spectrum of *m*-coumaric acid (**95)** at *m/z* 163.0389 (163.0381-163.0397) (mass tolerance 5 ppm) (for numbers and fragmentation patterns, see Table 1).

**Figure S29.** (-) ESI-MS/MS spectrum of vanillic acid (**96)** at *m/z* 167.0338 (167.0330-167.0346) (mass tolerance 5 ppm) (for numbers and fragmentation patterns, see Table 1).

**Figure S30.** (-) ESI-MS/MS spectrum of *o*-coumaric acid (**97)** at *m/z* 163.0389 (163.0381-163.0397) (mass tolerance 5 ppm) (for numbers and fragmentation patterns, see Table 1).

**Figure S31.** (-) ESI-MS/MS spectrum of scopoletin (**98)** at *m/z* 191.0350 (191.0340-191.0360) (mass tolerance 5 ppm) (for numbers and fragmentation patterns, see Table 1).

**Figure S32.** (-) ESI-MS/MS spectrum of salicylic acid (**99)** at *m/z* 137.0230 (137.0223-137.0237) (mass tolerance 5 ppm) (for numbers and fragmentation patterns, see Table 1).


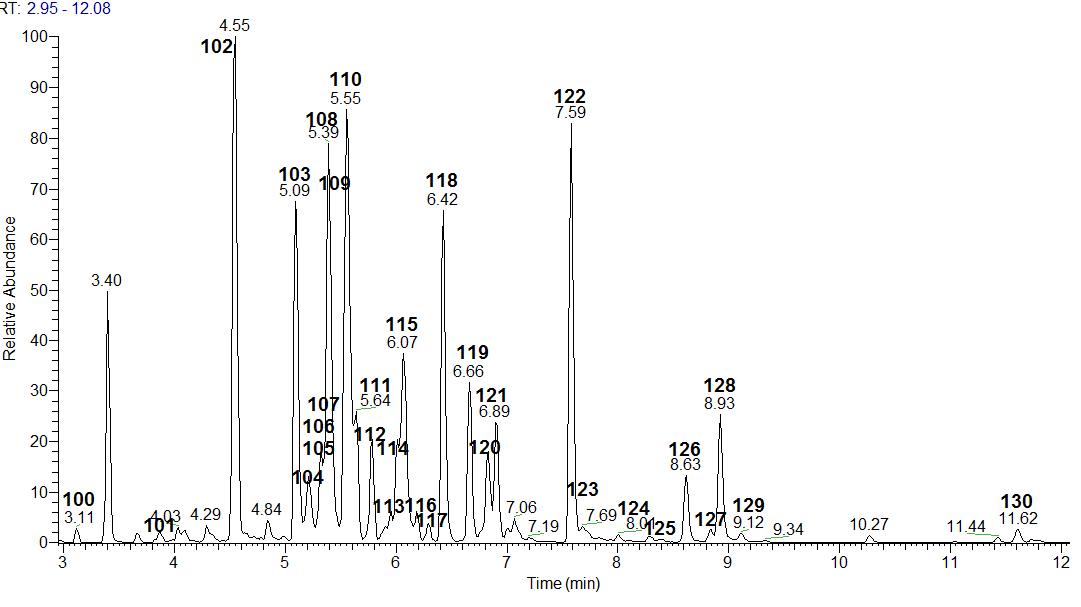


**Figure S33.** Extracted ion chromatogram (EIC) of flavonoids. EIC was proceed with mass tolerance of 5 ppm (for numbers and fragmentation patterns, see Table 1).

**Figure S34.** (-) ESI-MS/MS spectrum of rutin (**103**) at *m/z* 609.1464 (609.1434-609.1494) (mass accuracy 5 ppm) (for numbers and fragmentation patterns, see Table 1).

**Figure S35**. (-) ESI-MS/MS spectrum of luteolin 7-O-glucoside (**108**) at *m/z* 447.0933 (447.0911-447.0955) (mass accuracy 5 ppm) (for numbers and fragmentation patterns, see Table 1).

**Figure S36**. (-) ESI-MS/MS spectrum of nepetin 7-O-glucoside (**110**) at *m/z* 477.1038 (477.1014-477.1062) (mass accuracy 5 ppm) (for numbers and fragmentation patterns, see Table 1).

**Figure S37**. (-) ESI-MS/MS spectrum of kaempferol 3-*O*-rutinoside (**111**) at *m/z* 593.1512 (593.1014-593.1542) (mass accuracy 5 ppm) (for numbers and fragmentation patterns, see Table 1).

**Figure S38**. (-) ESI-MS/MS spectrum of isorhamnetin 3-*O*-rutinoside (**112**) at *m/z* 623.1618 (623.1587-623.1649) (mass accuracy 5 ppm) (for numbers and fragmentation patterns, see Table 1).

**Figure S39**. (-) ESI-MS/MS spectrum of apigenin 7-*O*-glucoside (**112**) at *m/z* 431.0984 (431.0962-431.1006) (mass accuracy 5 ppm) (for numbers and fragmentation patterns, see Table 1).

**Figure S40**. (-) ESI-MS/MS spectrum of luteolin (**122**) at *m/z* 285.0405 (285.0391-285.0419) (mass accuracy 5 ppm) (for numbers and fragmentation patterns, see Table 1).

**Figure S41**. (-) ESI-MS/MS spectrum of quercetin (**123**) at *m/z* 301.0354 (301.0339-301.0369) (mass accuracy 5 ppm) (for numbers and fragmentation patterns, see Table 1).

**Figure S42**. (-) ESI-MS/MS spectrum of nepetin (**124**) at *m/z* 315.0510 (315.0494-315.0526) (mass accuracy 5 ppm) (for numbers and fragmentation patterns, see Table 1).

**Figure S43**. (-) ESI-MS/MS spectrum of kaempferol (**126**) at *m/z* 285.0405 (285.0391-285.0419) (mass accuracy 5 ppm) (for numbers and fragmentation patterns, see Table 1).

**Figure S44**. (-) ESI-MS/MS spectrum of apigenin (**127**) at *m/z* 269.0455 (269.0442-269.0468) (mass accuracy 5 ppm) (for numbers and fragmentation patterns, see Table 1).

**Figure S45**. (-) ESI-MS/MS spectrum of isorhamnetin (**129**) at *m/z* 315.0510 (315.0494-315.0526) (mass accuracy 5 ppm) (for numbers and fragmentation patterns, see Table 1).

**Figure S46**. (-) ESI-MS/MS spectrum of genkwanin (**130**) at *m/z* 283.0612 (283.0598-283.0626) (mass accuracy 5 ppm) (for numbers and fragmentation patterns, see Table 1).
